# Variation in Tropical Reef Symbiont Metagenomes Defined by Secondary Metabolism

**DOI:** 10.1371/journal.pone.0017897

**Published:** 2011-03-22

**Authors:** Mohamed S. Donia, W. Florian Fricke, Jacques Ravel, Eric W. Schmidt

**Affiliations:** 1 Department of Medicinal Chemistry, University of Utah, Salt Lake City, Utah, United States of America; 2 Institute for Genome Sciences, Department of Microbiology & Immunology, University of Maryland School of Medicine, Baltimore, Maryland, United States of America; Netherlands Institute of Ecology, Netherlands

## Abstract

The complex evolution of secondary metabolism is important in biology, drug development, and synthetic biology. To examine this problem at a fine scale, we compared the genomes and chemistry of 24 strains of uncultivated cyanobacteria, *Prochloron didemni*, that live symbiotically with tropical ascidians and that produce natural products isolated from the animals. Although several animal species were obtained along a >5500 km transect of the Pacific Ocean, *P. didemni* strains are >97% identical across much of their genomes, with only a few exceptions concentrated in secondary metabolism. Secondary metabolic gene clusters were sporadically present or absent in identical genomic locations with no consistent pattern of co-occurrence. Discrete mutations were observed, leading to new chemicals that we isolated from animals. Functional cassettes encoding diverse chemicals are exchanged among a single population of symbiotic *P. didemni* that spans the tropical Pacific, providing the host animals with a varying arsenal of secondary metabolites.

## Introduction

Marine invertebrates such as sponges, ascidians, and mollusks are sources of abundant and diverse secondary metabolites, many of which are drugs or drug leads [1], [2]. The metabolites impact marine environments, and many have defined biological roles, for example helping animals to evade predation on coral reefs [3]. A major unanswered question has been what controls the distribution and synthesis of these animal compounds across the oceans. Marine invertebrate natural products are often very sporadic in their occurrence, and chemistry varies between seemingly identical samples, even from animals living next to each other [4]. It has been shown that a number of marine invertebrate natural products are actually synthesized by symbiotic bacteria [5], [6], [7], which is especially striking because some of these “bacterial” molecules may be critical for survival of hosts.

Secondary metabolism represents one of the most common and varied features of bacterial genomes [8], [9]. Animals harness this diverse bacterial metabolism for their survival, and it is increasingly apparent that this is a widespread phenomenon on both land and sea [10]. For example, in wasps a symbiotic bacterial species synthesizes a mini-library of antibiotic secondary metabolites to protect the animal larvae from bacterial infection [11]. However, very little is known about how animals obtain these defenses or about what controls variation in defensive chemicals found in animals. In cultivated bacteria, even in closely related strains chemistry is quite variable, but these strains have been removed from their environment, so that no immediate environmental relevance can be discerned. In addition, in cultivated bacteria there are usually many mutations between strains, so that precise genomic changes underlying chemical variation cannot be readily determined. By contrast, symbiosis affords an opportunity to examine immediately the effects of genomic changes in the real environment, and very precise genomic changes can be identified [10].

**Figure 1:**
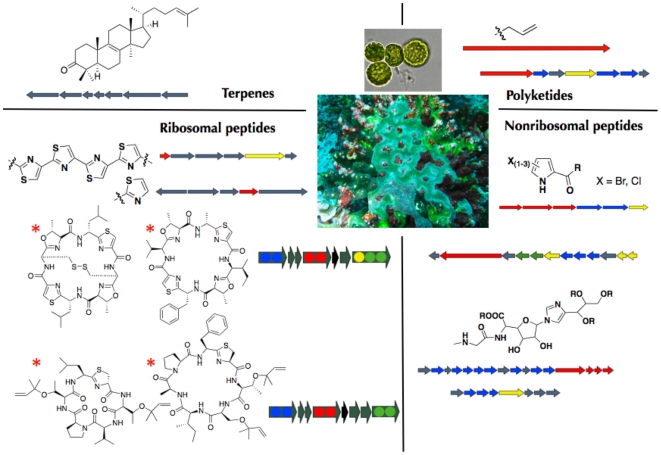
Secondary metabolic pathways from three *Prochloron* metagenomes. Center: *Prochloron* cells (top) from the ascidian *L. patella* (bottom). Surrounding are ten pathway types identified, comprising 15 distinct biosynthetic gene clusters in the metagenomes of P1, P2 and P3. Compounds that have been chemically isolated are indicated by an asterisk; the remainder are genome-based predictions.

For example, about 1000 natural products have been isolated from marine ascidian animals, including more than 60 cyclic peptides of the cyanobactin class that form large families of related compounds [12]. These potently cytotoxic compounds are often abundant components of the biomass, to the point that they are the major constituents of whole animal extracts. In addition, some species of ascidians house uncultivated cyanobacterial symbionts, *Prochloron didemni*, which form near monocultures in whole animals that can be readily observed by eye because of the resulting bright green color of the hosts (Figure 1) [13]. *P. didemni* is important to both the primary and secondary metabolism of host ascidians, and in some cases it has been shown to be essential for ascidian survival. *P. didemni* transfers fixed carbon and recycles nitrogen waste for the host ascidians, allowing the animals to survive in low-nutrient, high-light intensity environments [14]. We previously demonstrated that *P. didemni* synthesizes cyanobactin secondary metabolites isolated from whole ascidians and that bacterial strain variation underlies the varied “ascidian” natural products (Figure 1) [6], [15], [16], [17].

The *P. didemni*-ascidian system thus afforded a natural laboratory to study the precise genomic basis of symbiosis and pathway variation in a native context. The symbiotic bacteria are transmitted via an ascidian species-specific combination of horizontal and vertical transmission, allowing the bacteria to specialize within their ascidian hosts while potentially exchanging genetic information between individuals and even between other species in the ocean [18], [19]. Ascidian colonies are able to divide and fuse in just a few days [20], possibly facilitating such exchange. *P. didemni* inhabits many different species of ascidians that occupy different habitats [13]. Here, we apply metagenomic sequencing and chemical analysis to examine variation over whole genomes across the tropical Pacific. Three samples containing *P. didemni* were deeply sequenced, providing high-resolution data of the genomic changes across the tropical Pacific and defining three types of precise genomic changes that lead to libraries of natural products in animal-bacteria associations. We show that the observed strain variation is largely focused in sporadically distributed secondary metabolic gene clusters, providing the animal-bacterial assemblages with a variable arsenal of chemical products in an otherwise conserved genomic background. An additional 21 samples from diverse locations and habitats were further examined by PCR, verifying these trends across a vast swathe of the Pacific Ocean. Chemical analysis confirmed the genomic observations, leading us to discover that adjacent, identical colonies vary in their symbiotic and chemical content. This observation provides a new paradigm for natural product discovery and reveals a natural combinatorial laboratory for the synthesis of diverse chemicals harnessed by the animal hosts.

## Results

### Metagenome sequencing

We obtained didemnid ascidians from different habitats across the tropical Pacific, including Palau, Papua New Guinea, the Solomon Islands, and Fiji. Three samples of the ascidian *Lissoclinum patella*, collected on shallow outer-reef ledges, were used for metagenome sequencing: P1 (Palau), P2 (Fiji), and P3 (Solomon Islands). An additional 21 samples of diverse species and locations were further analyzed by a PCR-based approach. Because *P. didemni* is resistant to cultivation and constitutes over 95% of the host-associated microbes in some cases, preserved samples were examined using molecular methods. The metagenome of P1 was analyzed by Sanger sequencing and 454 pyrosequencing supplemented with fosmid library end-sequencing while P2 and P3 were analyzed using 454 pyrosequencing and fosmid end-sequencing. These methods allowed the genome of *P. didemni* P1 to be assembled into 39 scaffolds with 98 physical gaps, with a total sequence length of ∼6,035 kbp. P2 and P3 were sequenced to ∼90x and ∼14x coverage (based on contigs ≥1 kbp) and assembled with N50 sizes of 18.5 kbp and 7.7 kbp, respectively, into smaller fragments that are readily mapped to P1 (File S1).

### Genome analysis and secondary metabolism

Detailed bioinformatics methods were used to ensure the source and quality of the *P. didemni* sequence (see Materials and Methods). To determine whether the three sequenced samples could be composed of populations of multiple individual *Prochloron* strains with genomic inter-strain variations we examined several genetic loci in detail, including ribosomal RNA clusters, the *Prochloron*-specific chlorophyll *a* oxidase (*cao*) gene and all 15 secondary metabolite gene clusters. These genetic loci were examined at the read level and searched for genetic variation within the samples and changes in the coverage compared to the genome average. In all examined cases, we found no indication of multiple genomic variants within any of the three samples, supporting that only one major *Prochloron* genotype is present in each sample. The *cao* gene is a housekeeping gene specific to *P. didemni* that is essential for photosynthetic cofactor biosynthesis [21].

**Figure 2:**
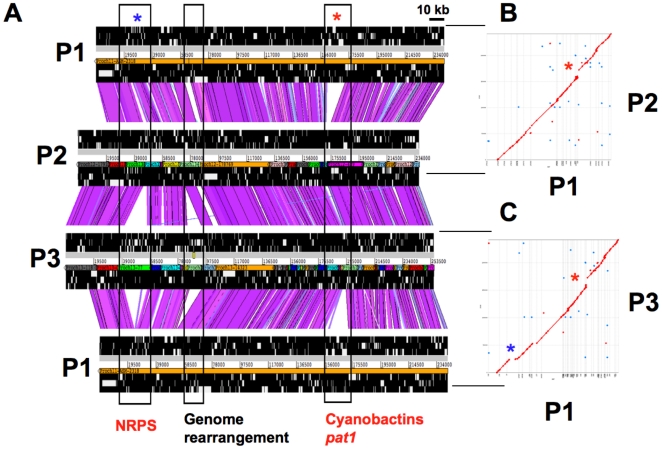
Variations in secondary metabolic gene clusters constitute the major difference between the *P. didemni* genomes. a) The Artemis Comparison Tool (ACT) was used to visualize pair-wise comparisons between contig ASM-2318 from P1 and corresponding contigs from P2 and P3 [44]. Violet color indicates ≥ 96% nucleotide identity while light blue color indicates 90–96% nucleotide identity. Two of the three differing regions marked by black boxes differ solely due to natural product gene clusters. This pattern is reproduced throughout the 3 metagenomes. b) and c) Dot Plot representations of NUCmer comparisons showing synteny between the same genetic regions as in A [45].

Complete draft genome sequences of all predicted contigs from P2 and P3 were largely identical to that of P1, with >97% DNA sequence identity across ∼90% of the P1 chromosome (Figure 2). The remaining ∼10% of these genomes is characterized by the presence of numerous, highly variable paralogous genes (such as multiple similar copies of ankyrin repeat-containing genes) containing multiple repetitive elements as well as a set of genes with no assigned function. Among the genes for which functions could be called, nearly all were involved in secondary metabolism (116 out of 126 assignable differences in pairwise comparisons). We also observed that individual sequence regions of interest appeared to be syntenic, leading us to propose that the entire genomes were largely identical and syntenic, with the exception of repeats, secondary metabolism genes, and a small number of other genes. This synteny was confirmed by numerous experimental methods defined in Materials and Methods.

**Figure 3:**
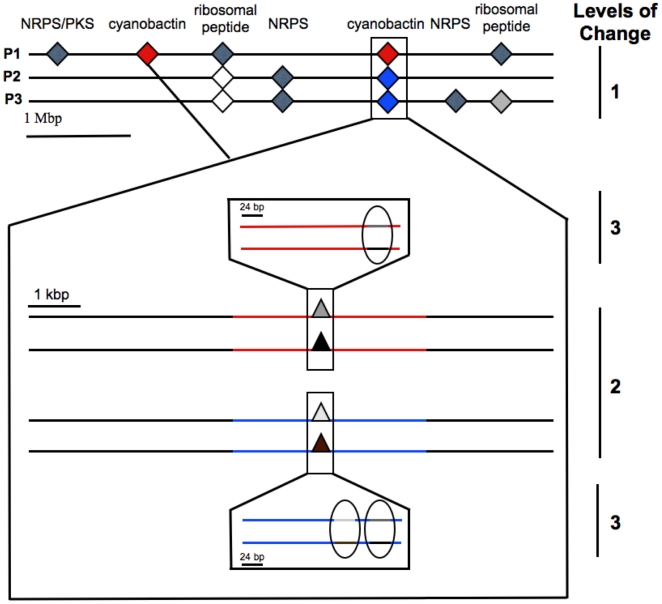
Schematic representation of the different levels of changes in functional cassettes in *P. didemni* genomes. Three levels can be observed: 1. Presence or absence of a gene cluster in the genome. Homologous gene clusters are >99% identical between strains; 2. Swaps of function (heterocyclization to prenylation) within otherwise >99% identical gene clusters; 3. Functional point mutations in gene clusters (for example in product-coding sequences of ribosomal peptides).

We analyzed the secondary metabolic gene content of P1, P2, and P3, searching for polyketide synthases, terpene synthases, and ribosomal and nonribosomal peptide pathways. Representatives of all four pathway types were identified in the genomes, leading to a total of ten biosynthetic gene families in 15 distinct clusters (Figure 1). A complete analysis of these clusters was performed, enabling prediction of likely products in most cases (Materials and Methods and File S1). Only three of these clusters are shared between all three strains, while the remainder are found only in one or two of the strains. In all cases where a cluster was found in more than one strain, the genes are essentially identical (>99–100% DNA sequence identity) and are present in conserved locations on the chromosomes. The chromosomal location could be assessed in the genome-sequenced strains and also for five specific pathways that were examined across an additional 21 samples that were examined by PCR (see below). We identified three trends, which to the best of our knowledge have not been previously observed at high resolution (Figure 3): 1) presence or absence of individual gene clusters in identical chromosomal locations, leading to the presence or absence of whole groups of natural products in ascidians; 2) crossovers within pathways leading to new functionality within very similar natural product types, all in identical chromosomal genetic backgrounds; and 3) hypervariable changes in short (<30 base pair) cassettes whose substitution leads to wholly different natural products in otherwise identical DNA sequence environments.

### Analysis of secondary metabolic gene clusters

Three nonribosomal peptide synthetase pathways were identified: *nkd*, *pyr*, and the previously reported *prn* [15]. *nkd* is predicted to encode a new compound related to the nikkomycins, which are normally associated with actinomycetes [22]. *pyr* is related to halogenated pyrrole pathways [23], while the product of *prn* cannot be accurately analyzed because of a noncanonical gene architecture. *nkd* was found only in P3, although a residual methyltransferase gene from the pathway was identified in P1 at the same chromosomal location. *pyr* was found in P2 and P3, while *prn* was found only in P1. Two polyketide synthase genes were identified: one of these is potentially part of the *prn* cluster and is found in P1 only, while the other, *gaz*, is found in all 3 strains. In these strains, *gaz* is identical except for a variable copy number of a small repeating sequence element. The *gaz*-encoded polyketide synthase is very similar to the C-terminus of the last polyketide synthase in the curacin cluster [24], [25]. We predicted that this gene should synthesize a terminal olefin hydrocarbon. Recently, cyanobacterial hydrocarbon biosynthesis was defined, and this type of pathway would widen bacterial hydrocarbon synthesis to include terminal olefins [26],[27]. A terpene synthase gene cluster that should lead to bacterial lanosterone biosynthesis is also present in all three strains [28].

The nonribosomal, polyketide, and terpene genes are discretely either present or absent in identical chromosomal locations in all three genome-sequenced strains, and when they appear their DNA sequences are essentially identical, although sometimes various numbers of nearly identical repetitive elements flank the gene clusters. The analysis of ribosomal peptide natural product biosynthesis is relatively more complicated. Four types of ribosomal peptide pathways were identified, with only one class being present in all three strains. In addition, where classes overlap between two strains, the genes encoding modifying enzymes are essentially (>99%) identical, while small portions of the precursor peptide genes are hypervariable, so that the actual natural products differ between strains. Although the phenomenon of bacterial precursor peptide variation is becoming more widely appreciated [29], [30], [31], *P. didemni* provides the first case where such precisely defined differences have been observed.

In ribosomal peptide pathways, a precusor peptide is modified by enzymes to produce natural products, such as the well-known lantibiotics and microcins [30], [32], The precursor peptide contains a leader sequence, which directs the peptide to enzymes and which is often later removed by proteases, and a core sequence, which encodes the mature natural product and which is the substrate for enzyme modification. In the four *P. didemni* ribosomal pathways, the core sequences are hypervariable, while the leader sequences and all of the flanking genes are nearly 100% identical.

Two new ribosomal pathways were discovered in this analysis: *hyd* and *tom*. While *hyd* is present in all three strains, *tom* is only present in P1 and P2. *hyd* was predicted to synthesize natural products similar to virenamides, which have been isolated from *Prochloron*-bearing ascidians [33], while *tom* is a streptolysin S-like cluster of the so-called TOMM group [29], [34]. Although the predicted modifying genes are identical when present and the clusters occupy identical genetic regions, the core sequences are hypervariable.

We previously reported an additional two classes of ribosomal peptide pathways, but with this report new genome-scale evolutionary analysis is available providing new insights into pathway evolution. These two classes, *pat* and *tru*, lead to synthesis of cyanobactin natural products, which are small, N-C macrocyclic peptides isolated from ascidians and free-living cyanobacteria, among other organisms (Figure 1, lower left) [6], [17]. *pat* leads to compounds that are also heterocyclic at cysteine, serine, and threonine. By contrast, *tru* products are heterocyclic only at cysteine and instead are prenylated at serine and threonine. Both product groups, which are potent cytotoxins, are abundantly concentrated, at up to ∼1% dry weight, in whole ascidians. As previously reported, *pat* and *tru* are essentially identical in most of the ∼11 kbp operons, comprising the 5′- and 3′-regions of the pathways. In the central ∼4.5 kbp regions of the operons, all genes are homologous, but their DNA sequence identity lies between 77% and 25%. This crossover region is solely responsible for imparting the change in prenylation and oxidation states, as demonstrated previously by pathway expression in *E. coli* [6], [16], [17].

*pat* is only present in P1. However, there are 2 nearly identical copies of *pat* that are distantly located on the P1 chromosome. In *pat1*, a precursor peptide encodes the synthesis of the “ascidian” natural products patellamides C and A. In *pat2*, the precursor peptide encodes the products patellamides C and ulithiacycliamide (in place of patellamide A). These entire ∼11 kbp pathways have identical DNA-sequences except for the 24-base pair cassettes encoding patellamide A or ulithiacyclamide, which are only 46% identical [16].

*tru* is present in both P2 and P3 in a single copy, in the same location as the *pat2* pathway in P1 (Figure 3). The flanking regions are identical to *pat*, as noted above, and only the central prenylating/oxidizing region differs between *pat* and *tru*. From whole-genome sequencing, it is apparent that the ∼11 kbp *tru* clusters from P2 and P3 are also essentially identical, with two exceptions. Like *pat*, they encode different natural products in hypervariable core sequences, including the anticancer lead compound, trunkamide [17]. Unlike *pat*, where the clusters are identical along their lengths, the two *tru* pathways have recombined with *pat* at least twice, leaving scars in slightly different locations. In P2 and P3, the integration site of the *tru*-specific region differs by several base pairs in the 5′-region, and by nearly 300 base pairs in the 3′-region. This is striking given that the samples were obtained >1000 km apart in the ocean and can only be reasonably explained by at least two separate recombination events.

### Sporadic variation of secondary metabolism

Based on comparisons of predicted *P. didemni* contigs, P2 and P3 seem to be phylogenetically slightly more related to each other (98% identity) than to P1 (97% identity). Nonetheless, the secondary metabolic pathways vary just as much between P2 and P3 as they do between either strain and P1. Therefore, it seemed that the variation in pathways between these strains could be largely a result of gene loss from an ancestral strain containing all of the pathways, or alternatively horizontal transfer from non-*Prochloron* strains could account for the differences. To test these hypotheses, 21 additional samples from a >5500 km transect of the Pacific were analyzed chemically and by PCR targeting the integration loci for the presence/absence of biosynthetic genes (File S1). These samples included the microbial content of taxonomically diverse ascidian species and multiple replicates of *L. patella*, which were collected at different times (2002, 2003, 2005, 2006, and 2007) and over an extensive transect. Because the exact site of pathway insertion could be identified from genome sequencing, excellent PCR controls were available: primers were designed for pathway genes and to amplify across flanking regions, providing positive PCR products for either the presence or absence of most pathways, with a few exceptions (File S1). However, in the cases where the pathways are flanked by long repetitive elements, primer design to amplify across the pathway was impractical.

**Figure 4:**
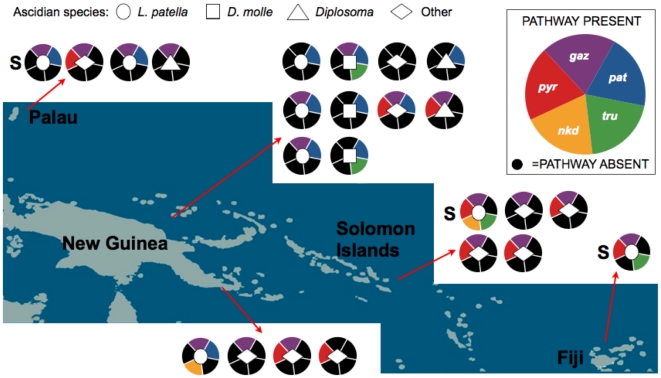
Sporadic distribution of ascidian-associated secondary metabolic genes across the tropical Pacific. Twenty-four samples collected over a 5,500 km transect in the tropical Pacific were screened for presence/absence of genes for five biosynthetic pathways. Each circle represents an individual ascidian sample. The shape inside the circle indicates the species of the *Prochloron*-harboring ascidian, as shown in the key above. Colors within the circle represent the presence of individual biosynthetic pathways, which are >99% identical between samples, while black indicates the absence of a pathway from the sample. **S** represents samples for which whole genome sequence was obtained.

Using the above strategy, there were no cases in which both the presence and absence reactions were positive, confirming the robustness of the controls. This analysis also ruled out the possibility that pathways are inserted in different genomic contexts, which would be clearly revealed by positive amplification with the pathway internal primers and negative amplification with the flanking primers. Strikingly, there is no pattern to the presence or absence of secondary metabolite genes in the 24 samples studied (Figure 4). From each ascidian taxonomic group, time point and location, there are representatives that either contain or lack a specific pathway. In the cases where pathway products are known, the presence or absence of a certain pathway relates directly to the chemicals isolated from the whole ascidian sample.

These results are inconsistent with the two simple hypotheses proposed. Gene loss alone, although it clearly occurs as exemplified by a residual methyltransferase in the *nkd* group, cannot account for the observed sporadic distribution. Horizontal acquisition from more distantly related strains also cannot explain the sporadic occurrence of these pathways, since in that case they would not be identical, nor would they be likely to always lie in precisely the same positions. The results are also at odds with the idea of core and dispensable secondary metabolic genes in individual bacterial species, since no pathway is universal. Instead, we propose that this natural gene variation results from horizontal exchange within nearly identical *Prochloron* strains. At least in didemnid ascidians in the tropical Pacific, *Prochloron* strains from multiple species should be considered as a single population that can exchange biosynthetic capacity, while nearly all other core genes are highly conserved.

### Colony variation in didemnid ascidians

These differences in pathway types impact the chemistry of whole ascidian animals found in tropical reef environments. In the tropical Pacific ascidians of the genus *Didemnum molle* often form numerous, adjacent small colonies that are known to fuse and divide in the course of just a few days [20]. Since we knew from genome comparison that *P. didemni* varied in natural product content, we wished to determine whether even adjacent *D. molle* colonies might exhibit this variation. If so, fusion of ascidian hosts might provide a natural laboratory for recombination and diversification of ascidian compounds.

**Figure 5:**
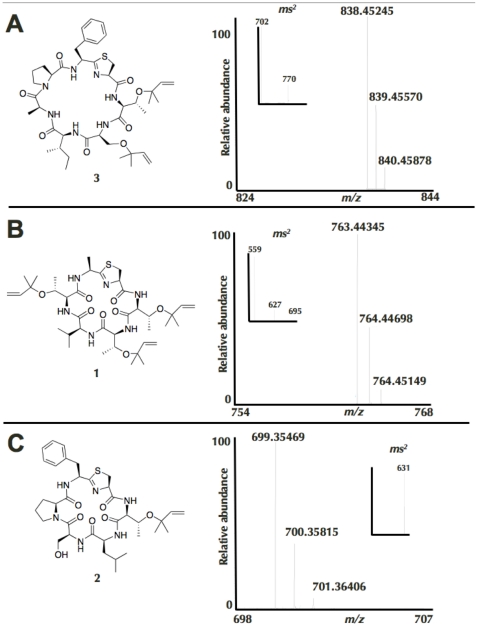
New prenylated cyanobactins discovered by comparative analysis. A control known compound, trunkamide (**3**, panel a), was subjected to high-resolution mass spectrometry analysis. Using tandem mass spectrometry, both one and two isoprene side chains (C_5_H_8_, *m/z* = 68.0626) were eliminated from this molecule. In comparison, two new compounds, mollamide D (**1**) and mollamide E (**2**) from sample 03-002, also exhibited the same pattern (panels b and c). The high-resolution results corresponded both to the predicted masses of the compounds and to the predicted loss of isoprene.

*D. molle* samples 03-002, 03-009 and 03-011 were collected at adjacent locations in Madang Bay, Papua New Guinea. Although they appeared very similar to each other, each had a unique pattern in respect to the five biosynthetic pathways examined, based on PCR analysis (Figure 4). We sequenced these PCR products and determined that *D. molle* samples 03-002 and 03-011 contain *tru* pathway variants leading to previously unknown natural products. These products are encoded on similar but not identical precursor peptides (File S1). We wanted to examine if this genomic difference directly translated to the chemical content of the animal colonies. Precise chemical analysis of a single colony confirmed that 03-002 contained two new cyanobactins of the rare prenylated group, which often has anticancer activity (Figure 5). The new cyanobactins, mollamide D (**1**) and E (**2**), and their biosynthetic genes were not found in neighboring ascidian colonies. These results indicate that three very closely related, neighboring and possibly fusing ascidian colonies contain the three examples of evolutionary changes described above, reinforcing the impact of sporadic diversity on animals in the environment. In addition to chemical cyanobactin differences, each of these samples harbors a different reservoir of gene cluster types. When similar ribosomal peptide pathways are found, the precursor peptides differ, leading to different products. Together, these genetic changes result in completely different chemical compositions.

## Discussion

This study demonstrates the power of analyzing biosynthetic genes from closely related symbionts for the study of pathway evolution as well as for drug discovery. Two new cyanobactins were identified in chemical extracts, and three types of pathway evolution were observed at a very fine scale. The phenomenon of secondary metabolite variation within and between strains is very well known. Pathway variation has been extensively studied in actinomycetes at the genomic level, although in general too many changes in gene content, conservation, and order are present to draw the conclusions apparent from this study [35]. Similar myxobacterial strains have been examined chemically, showing sporadic presence/absence of pathway types, but it is currently unclear how this will manifest at the genome level [36]. We previously described hypervariability within *pat* and *tru* precursors, and more recently extreme hypervariability in ribosomal precursor peptide sequences has been described in a number of additional bacterial strains, using bacteria that are not as closely interrelated as in this study [37]. However, in no case so far is the DNA sequence identical except in the very small core region of these peptides. The closely related symbionts, *P. didemni*, provide a laboratory in which three levels of precisely observable genomic change lead to an arsenal of variable natural products.

We speculate that this sporadic pattern might be driven by the processes controlling the symbiosis and a need to evolve new defense strategies for the host. As one example, the patellamides, trunkamide, and relatives comprise up to 1% of the dry weight of whole animal-bacterial assemblages, and in some cases they exhibit low nanomolar toxicity to human cell lines. Therefore, cyanobactins are potent enough and present in great enough quantity to influence the ascidian animal and, potentially, predators or competing bacteria, although this is a speculative role. Moreover, other pathways discovered in *P.didemni* are predicted to synthesize compounds active against possible competing microbes, such as the products of *pyr* and *nkd*. It is worth re-emphasizing here that the observed distribution was sporadic – that is, there was no pattern of co-occurrence, defying simplified attempts at defining roles for these compounds. Such simplified statements about natural products are unfortunately very common but serve to obscure the reality of extreme metabolite variation that is actually observed in nature. In this study, we show that natural products are distributed in a truly sporadic manner across a population and that it is the major variable feature in that population. Since the primary role of bacterial recombination is to repress diversity [38], we propose that the variation itself, and not the persistence of individual molecules, is the important factor. Instead of producing only a few compounds, this mechanism allows the population of *P. didemni* to obtain and combinatorialize diverse small molecules. In turn, access to these molecules would help the ascidians and bacteria to face the varying challenges that are actually encountered in nature. Ascidian fusion and fission takes place in the course of just a few days [20], allowing this selection to play out rapidly in response to new challenges.

It has long been anecdotally known that seemingly identical marine animals contain different natural products, even when they live adjacently. This has been especially true in the didemnid family ascidians, where neighboring colonies seem to have different chemistry. Here, we take a whole genome approach and demonstrate a new process of genetic exchange governing the distribution of secondary metabolites in the ocean. The sporadic variation hypothesis explains the observed variation of natural products in ascidians that harbor *Prochloron*, and similar types of pathway variation may impact distribution of other important marine natural products from ascidians, sponges, and other groups of invertebrates. Indeed, the Piel group recently described polyketide synthase genes and a housekeeping gene from bacterial symbionts of sponges [39]. Although the polyketide synthase genes were sometimes present and sometimes absent, a housekeeping gene was very nearly identical in samples harboring or missing the polyketide pathway. It thus appears that pathway variation within otherwise nearly identical symbionts may be a widespread phenomenon that governs the distribution of many marine invertebrate natural products. The resulting natural products play major roles in the environment, in physiological function, and in drug development. However, this trend is not universal: for example, it is likely that the bryostatin pathway in bryozoans does not follow this model [40].

Here, we show that analysis of symbiotic metagenomes provides precise resolution of pathway presence and evolution and may therefore be of use in informing synthetic biology. These results also help to explain the distribution of pharmaceutically important natural products from marine invertebrates and support continuing exploring a species for new natural compounds. Since colonies of otherwise identical ascidians can vary in the chemistry produced, there is potentially an enormous reservoir of undiscovered compounds in these organisms. This chemical diversity can be explored by simple PCR-based analysis, which can direct chemical efforts as exemplified by the discovery of mollamides D and E. Although ribosomal peptides afford a relatively simple example since the products are directly genetically encoded, as technologies improve this discovery method will be possible for other natural product pathway types as well. We provide proof-of-concept that individual, adjacent animal colonies differ in their natural product arsenal, implying a new model for discovery of marine natural products. We also report numerous orphan natural products clusters that are quite different from characterized pathways using just a few samples.

## Materials and Methods

### Ascidian samples

Samples were obtained from reef edges in Palau (P1, 2002), Fiji (P2, 2006), and the Solomon Islands (P3, 2006). To obtain DNA for sequencing, freshly collected *L. patella* was squeezed to release *Prochloron*, and DNA was immediately processed as described previously [15], [17]. For P2, the isolated DNA from the squeezed sample was combined with total DNA isolated from the whole *L. patella* sample (1∶1 mass ratio). Additional samples for PCR screening were obtained from Palau, Papua New Guinea, Solomon Islands, and Fiji in years 2002, 2003, 2005, 2006 and 2007. Habitats included channels, harbors, seagrass beds, reef tops and edges, walls, and seamounts, while organisms included several species in the genera *Lissoclinum*, *Didemnum*, and *Diplosoma*. Fosmid libraries with an average insert size of 40,000 bp were generated from samples P1 and P2 as previously described [15], [17]. Collection locations are defined in File S1. Samples were obtained with appropriate permits and agreements in place.

### DNA sequencing

Whole-genome sequence data for P1 were generated using Sanger sequencing as described previously [6]. 454 GS20 pyrosequencing was performed on sample P1 to increase coverage. Since DNA isolations from the squeezed samples yield a small fraction of non-*Prochloron* or eukaryotic host DNA (together ∼5%), a separation of *Prochloron* sequence reads was achieved using the Glimmer gene finding software [41]. All sequence reads from the P1 sample, including 107,589 Sanger reads and 1,285,077 454 reads, were assembled using Celera Assembler and a large number of the remaining sequence gaps closed through primer walking and PCR closure techniques. Samples P2 and P3 were sequenced using the 454 GS FLX Titanium series with a total number of 2,837,355 and 387,519 sequence reads, respectively. To separate *P. didemni* sequence contigs in the P2 and P3 samples from eukaryotic or other bacterial sequence contigs, the PhymmBL metagenomic classification tool was applied, which uses Interpolated Markov Models [42]. Since both samples were mainly composed of *P. didemni* genomic DNA, it was assumed that larger contigs would be almost entirely composed of *P. didemni* sequence reads. Consistently, PhymmBL classified these contigs as belonging to the phylum Cyanobacteria. To check whether PhymmBL could be optimized for the detection of *P. didemni* sequences, a new Interpolated Markov Model based on all contigs from the P2 sample larger than 10 kbp was built. Using this optimized model, no significant improvement of contig separation could be achieved. While contigs previously identified as *P. didemni* scored slightly better with the new model, it did not assign contigs that were previously not assigned to cyanobacteria to *P. didemni*. In addition, Sanger fosmid end sequences from each constructed library were obtained (5×96 well plates from P1 and 7×96 well plates from P2) with an average read length of 800 bp.

### Genome analysis

The P1 draft genome now consists of 39 scaffolds, i.e. ordered contigs that are connected through paired-end sequence reads spanning physical gaps between the contigs, with a total sequence length of ∼6,035,098 bp. The PhymmBL metagenomic sequence classification tool assigned 3,261 contigs and 1,964 contigs of the P2 and P3 samples, respectively, to the phylum Cyanobacteria, which together add up to a total size of 7,519,338 bp (31.5% of the total P2 metagenome) and 5,855,009 bp (82.0% of the total P3 metagenome). These contigs were considered *P. didemni* sequence and used for all following analyses. The fragmentation of the *Prochloron* draft genomes does not result from an insufficient sequencing coverage as revealed by more than 12x coverage in contigs ≥1 kb.

Overall genome comparison between P1, P2 and P3 showed large regions of synteny and average nucleotide identity of >97%. Techniques applied for comparative analyses included paired-end fosmid mapping from one genome onto the others, comparisons of large contigs (>50 kb) on the DNA and protein sequence level, and the identification of core gene sets shared between the three genomes. Despite this high degree of conservation, different secondary metabolic gene clusters were identified in all three genomes. In order to understand the genetic basis behind the variation in the secondary metabolome content, biosynthetic gene clusters including the surrounding genomic contexts were compared in detail.

### Demonstration of synteny in genomes

Numerous methods were used to show that the genomes were largely identical and syntenic. Although all three genome datasets are highly fragmented, contigs of a combined length of ∼500 kbp from all three genomes (∼8%) could be mapped and compared (Figure 2 and File S1). For example, P1 contig ASM-2318 is 241,607 bp in length. When comparing it to the P2 assembly, 22 contigs of different sizes mapped readily to it with no apparent synteny breaks except in a secondary metabolite gene cluster, *pat1*. When the same contig was compared to the more fragmented P3 assembly, 40 contigs mapped with only three apparent synteny breaks. Among those, two were related to the secondary metabolic gene clusters (*pat1* and *nkd*) while the third one appears to result from a genome rearrangement event or misassembly in P3. It should be emphasized that we can only be certain of synteny within the contiguous P2 and P3 regions that map to P1, while each contig end is a potential synteny break, since the contig order was not determined experimentally. To test whether the P2 contigs are arranged in the same order as in P1, we mapped the end sequences of 29 P2 fosmids with >99% DNA sequence identity to P1 contig ASM-2318. Indeed, the mapped paired-end sequence reads confirmed that the organization of P2 was identical to P1 over ASM-2318 (File S1). Similarly, P1 contig ASM-2352 (193,832 bp) was mapped to 21 contigs of P2 (no apparent major synteny breaks), and 29 contigs of P3 (only one synteny break of a region present elsewhere in P1 and P2, which could be due to genome rearrangement or misassembly) (File S1). Further, this assumption is supported by dot plots of nucmer comparisons of all contigs >5 kbp (P1: 83 contigs, P2: 342 contigs, P3: 347 contigs), which show no indication of within-contig synteny breaks (File S1). Precise locations could be identified where clusters involved in secondary metabolism were integrated into the *P. didemni* genomes; when a pathway was missing in a strain, the exact site of integration could be found because of highly conserved flanking genes.

### Identification of secondary metabolic genes in P1, P2, and P3

To identify secondary metabolic gene clusters in P1, a database was constructed by compiling protein sequences from GenBank of secondary metabolism biosynthetic genes of all types of interest from different organisms. These included nonribosomal peptide synthetases, polyketide synthases, cyanobactin synthetases together with all the auxiliary modifying enzymes including methyltransferases, oxidases, heterocyclases, and others. When this database was compared to the P1 assembly using tBLASTn [43], a few contigs were identified as hits and were analyzed in detail. Two polyketide synthase gene clusters, one nonribosomal peptide gene cluster, two cyanobactin gene clusters and two ribosomal peptide gene clusters were identified using this method. All identified hits were confirmed using the gene finding tool Glimmer and the gene annotation tool Manatee from The Institute For Genomic Research (TIGR) [41]. In addition, a terpene gene cluster was identified using Manatee.

To compare this set of gene clusters to the biosynthetic pool of P2 and P3, a similar but more complex pipeline was developed (File S1). First, the assembled whole metagenomic data sets from P2 and P3 were compared against the same secondary metabolite biosynthetic gene set (using tBLASTn) to identify matching contigs and unassembled sequence reads. These matching sequences were confirmed to originate from cyanobacterial species based on the taxonomic classification of all contigs and unassembled reads using the PhymmBL tool [42]. Matching sequences from cyanobacteria were then compared to paired-end sequences from fosmid libraries generated from DNA isolated from sample P2 and to assembled contigs from P1 to assure that they originated from *P. didemni* and not from any other cyanobacteria in the metagenome. For example, fosmid clones containing a new gene cluster on one end and a sequence with 97% percent identical to a P1 contig on the other end clearly originate in *Prochloron*. The newly identified secondary metabolite gene cluster-containing contigs that had no matches among fosmid sequences were compared to the longer P1 contigs using NUCmer and Artemis Comparison Tool (ACT) [44], [45]. In most cases, newly identified secondary metabolite gene clusters represented the only observed differences between the samples P2 or P3 and P1 in otherwise virtually identical (>97% DNA sequence identity) backgrounds. Using these methods, six additional gene clusters were identified in P2 and P3, encoding nonribosomal peptides, cyanobactins and ribosomal peptides.

### Detailed analysis of specific secondary metabolic genomic regions

A reverse pipeline to the one described above was developed (File S1). In this pipeline, large secondary metabolic contigs from P1 were used as a query and compared to the P2 and P3 metagenomic data sets. Identified hits were used for a pairwise NUCmer comparison against the original P1 contig. For a more detailed comparison method, identified contigs were concatenated manually to form a pseudomolecule, guided by information from NUCmer comparisons and paired-end fosmid mappings. ACT was used to visualize BLAST comparisons of the pseudocontig to the original P1 contig. This later comparison allowed the observation of differences as small as 1 kb between the compared contigs.

### PCR screening

Primer pairs were designed based on the identified secondary metabolite biosynthetic gene clusters in P1, P2 and P3. When gene clusters were present in one genome and absent in another (*pyr, nkd*), primers were designed to detect both genotypes (File S1). When gene clusters were present in all three genomes (*gaz*, *pat* and *tru*), primers were designed to only detect the presence of a pathway. All PCR products indicative of the presence of *pat* and *tru* were sequenced and verified. Reactions were optimized for each primer set and employed negative (sterile water) and positive controls.

### GenBank

All of the described data has been deposited in GenBank, accession numbers HQ407366-HQ407375.

### New cyanobactins

Ascidian samples were extracted with methanol and analyzed using high pressure liquid chromatography-electrospray-time of flight mass spectrometry (HPLC-ESI-TOF). Sample 03-002 was analyzed using FT-ICR MS to confirm the presence of two new cyanobactins. Sequence results from sample 03-002 showed that this sample should contain cyanobactins with sequences TTVTAC (mollamide D, **1**) and TLSPFC (mollamide E, **2**) (File S1). Sample 03-002 is a *D. molle* colony that is of insufficient size to perform nuclear magnetic resonance experiments. Therefore, we developed mass spectrometry methods that exploited the property of prenylation to definitively characterize the planar structures of the compounds, using the control compound trunkamide (**3**).

The predicted **1** and **2** were dominant in the HPLC traces of both TOF and FT mass spectrometry experiments (File S1). FTMS: **1**: *m/z*  = 763.44345; predicted 763.44223 for C_38_H_62_N_6_O_8_S (Δ = 1.6 ppm). **2**: *m/z*  = 699.35469; predicted 699.35342 for C_35_H_50_N_6_O_7_S (Δ = 1.8 ppm). (**3**): *m/z*  = 838.45245; predicted 838.45312 for C_43_H_63_N_7_O_8_S (Δ = −0.8 ppm).

Tandem mass spectrometry (ms^2^) of the known compound **3** using infrared multiphoton dissociation (IRMPD) led cleanly to loss of both one and two isoprene units without significant further fragmentation, providing a predictable pattern. Importantly, isoprene was lost with high resolution, providing extremely strong evidence of composition of matter. In tandem with sequencing data, this level of evidence is very high and similar to what is routinely reported in proteomics-based analyses. However, these data do not differentiate between the possible sites for placement of isoprene on **2**. IRMPD: **1**: -1 x isoprene: *m/z*  = 695.37764; predicted 695.37963 for C_33_H_54_N_6_O_8_S (Δ = −2.8 ppm); -2 x isoprene: *m/z*  = 627.31593; predicted 627.31703 for C_28_H_46_N_6_O_8_S (Δ = −1.7 ppm); -3 x isoprene *m/z*  = 559.25347; predicted 559.25443 for C_23_H_38_N_6_O_8_S (Δ = −1.8 ppm). **2**: -1 x isoprene: *m/z*  = 631.29075; predicted 631.29082 for C_30_H_42_N_6_O_7_S (Δ = −0.01 ppm). **3** (standard): -1 x isoprene: *m/z*  = 770.39055; predicted 770.39053 for C_38_H_5_N_7_O_8_S (Δ = −0.03 ppm); -2 x isoprene: *m/z*  = 702.32788; predicted 702.32793 for C_33_H_47_N_7_O_8_S (Δ = −0.07 ppm).

## Supporting Information

File S1 **Supporting figures and tables.** This file contains supporting figures and tables describing the samples used in this study, genome statistics, bioinformatics pipelines employed, genome synteny analyses, biosynthetic pathway analyses and prediction, PCR experimental design, list of primers, and mass spectrometry data.
(PDF)
