# Supplementary material for: Variation in Tropical Reef Symbiont Metagenomes Defined by Secondary Metabolism
Source: PLoS One. 2011 Mar 22;6(3):e17897. doi: 10.1371/journal.pone.0017897 (PMC3062557; doi:10.1371/journal.pone.0017897)
Supplement: File S1 — Supporting figures and tables. This file contains supporting figures and tables describing the samples used in this study, genome statistics, bioinformatics pipelines employed, genome synteny analyses, biosynthetic pathway analyses and prediction, PCR experimental design, list of primers, and mass spectrometry data. (PDF) [file pone.0017897.s001.pdf]

Supporting Information S1. Supporting figures and tables. This file contains supporting figures and tables describing the samples used in this study, genome statistics, bioinformatics pipelines employed, genome synteny analyses, biosynthetic pathway analyses and prediction, PCR experimental design, list of primers, and mass spectrometry data.

7 Supporting Tables

26 Supporting Figures

Figure S1. Pipeline for identification of *Prochloron* biosynthetic gene clusters in ascidian metagenomes. First, the gene cluster query is compared to the whole metagenomic data set using tBLASTn. Second, identified matching sequences are evaluated by the PhymmBL classification tool to ensure their origination from cyanobacteria. Third, identified contigs are compared to *Prochloron* sequences to verify their origination from *Prochloron*.

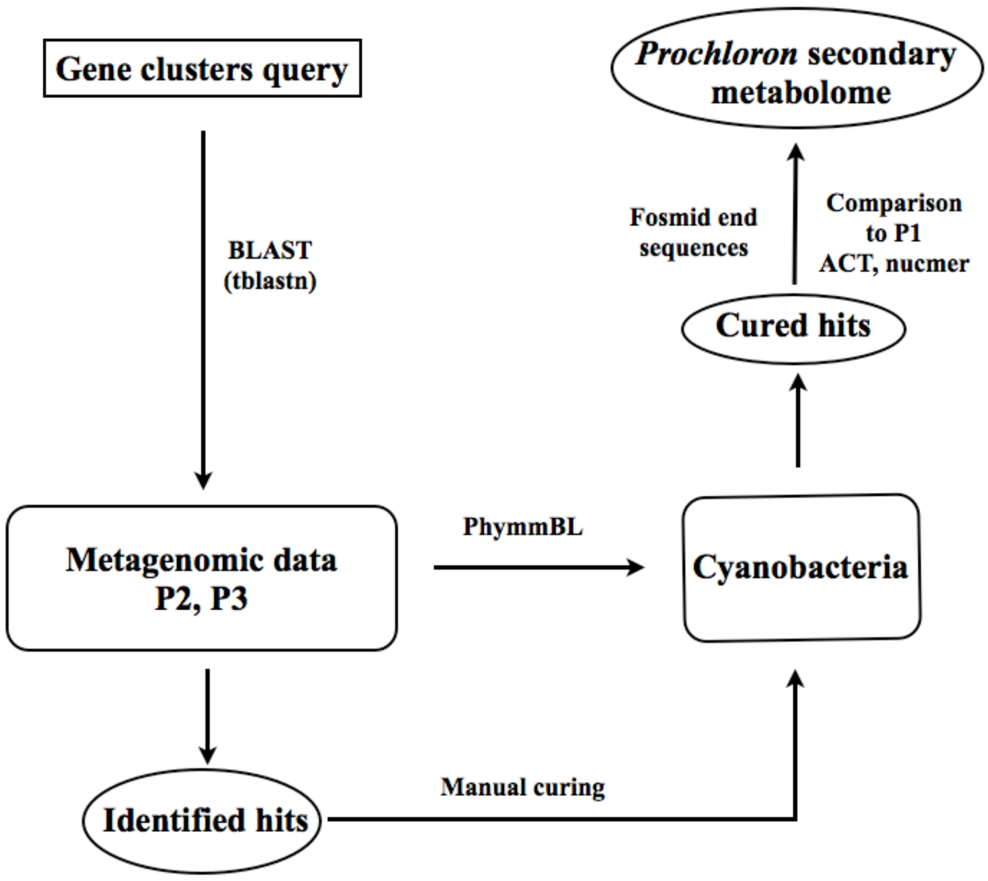

Figure S2. Pipeline for the comparison of long contigs involved in secondary metabolism. First, a selected P1 contig is used as a query for BLAST (blastn) against the assembled metagenomic data sets of P2 and P3. Identified contigs are then collected and use for NUCmer comparisons against the original P1 contig to examine synteny and coverage. In addition, the identified contigs are used to construct a pseudocontig using information from fosmid end-sequences and the generated Dot Plot from the NUCmer analysis. The generated pseudocontig is finally compared to the original P1 contig, and visually inspected for a more detailed analysis using ACT.

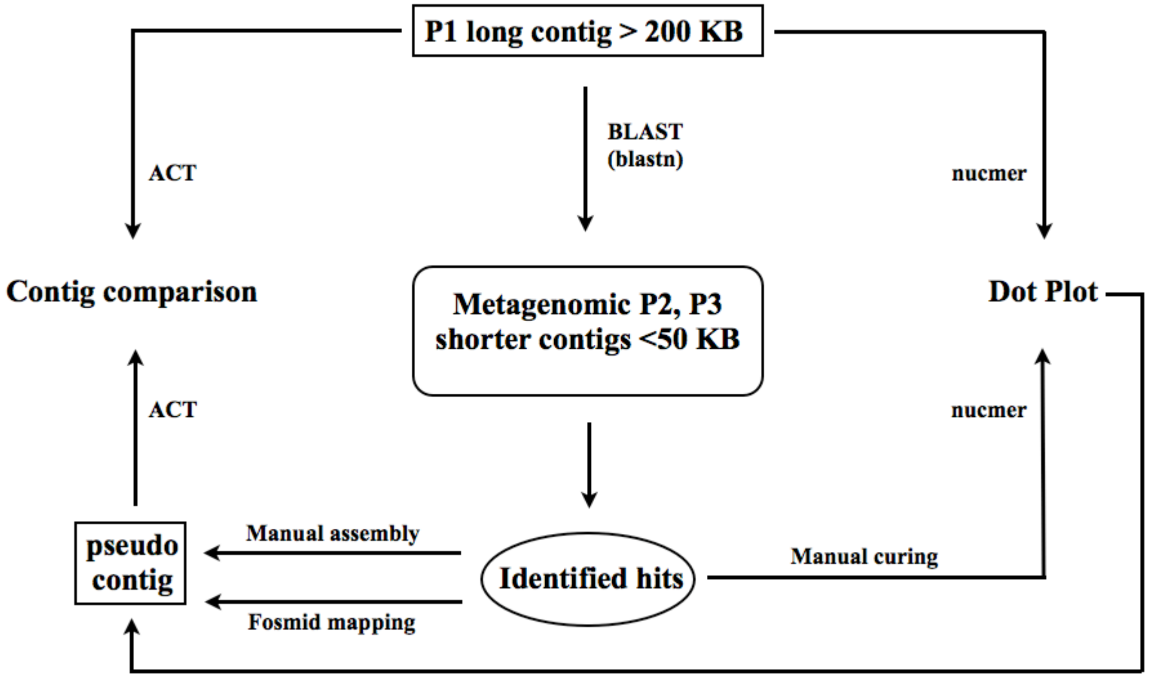

Figure S3. Analysis of contig ASM-2352. Using the pipeline described above, hits from a) P2 and b) P3 were obtained. These hits were then compared to contig ASM-2353 using NUCmer Dot Plots were generated by MUMmerplot. Total synteny and full coverage can be observed except for the rearranged region in P3 (arrow).

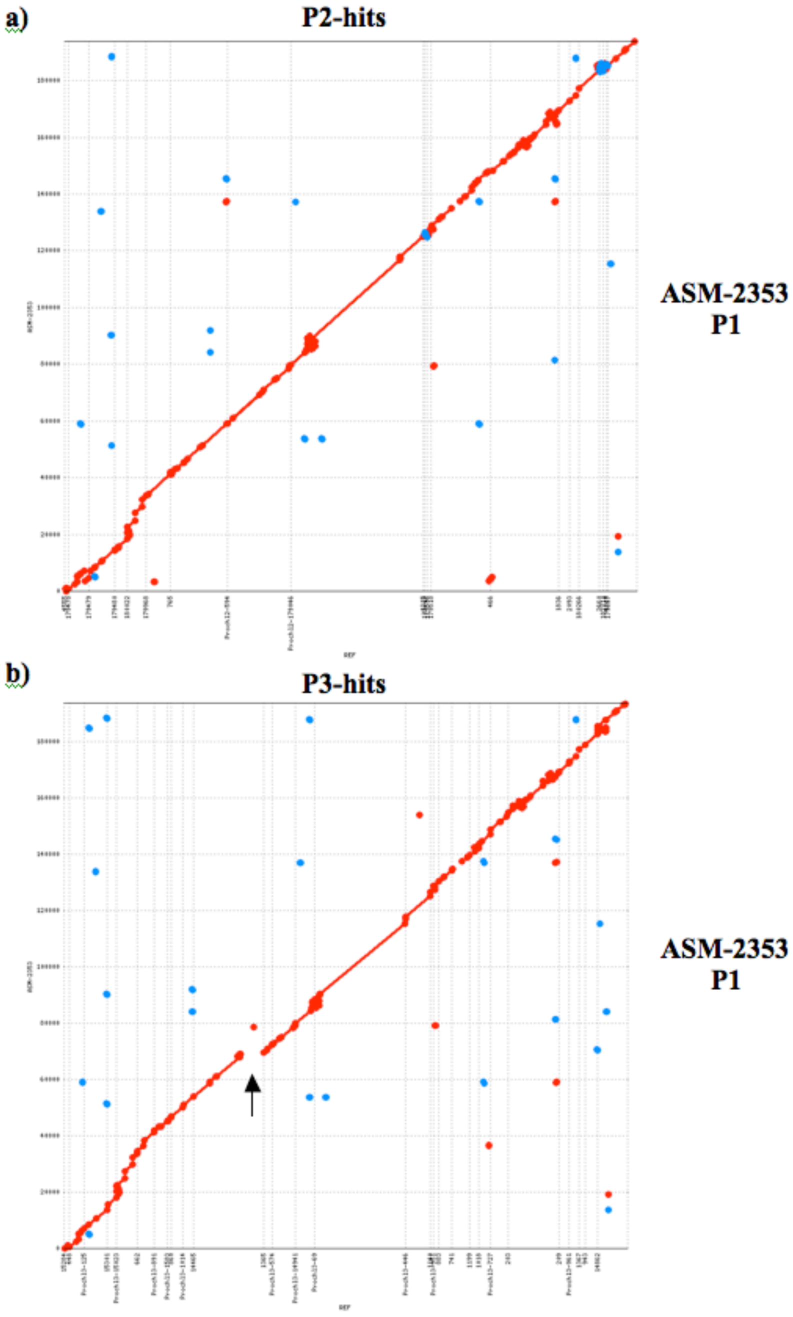

Figure S4. Analysis of contig ASM-2318. Using the pipeline described above, hits from a) P2 and b) P3 were obtained. These hits were then compared to contig ASM-2318 using NUCmer Dot Plots were generated by MUMmer Plot. Total synteny and full coverage can be observed except for the previously described regions harboring biosynthetic gene clusters.

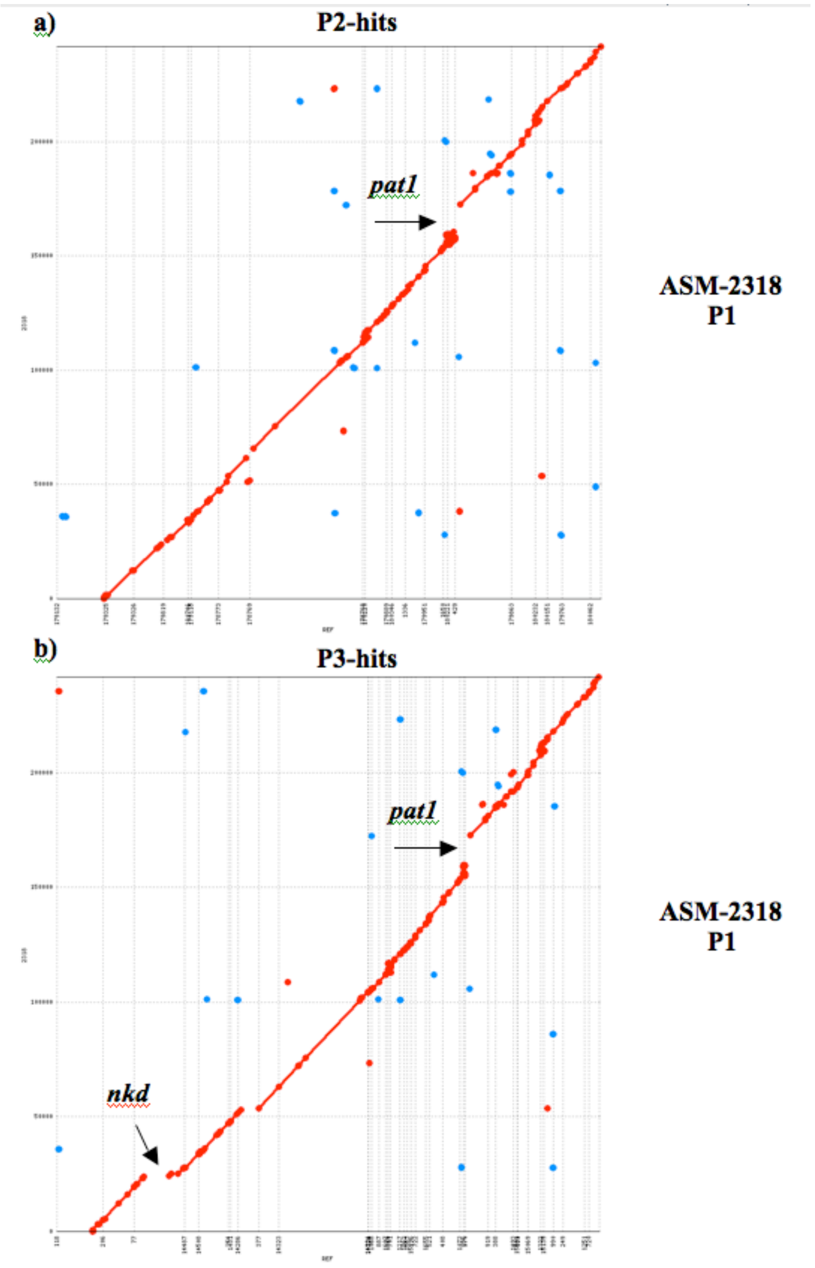

Figure S5. Fosmid mapping of contig-2318. Fosmids paired-end sequences were compared to the hits of ASM-2318 from the P2 metagenomic data set and used to direct the assembly of these shorter contigs into one pseudocontig. Horizontal lines indicate fosmids spanning the pseudomolecule where the two ends match the contig within the expected size of fosmid inserts. Numbers at the bottom are in 1000 bp.

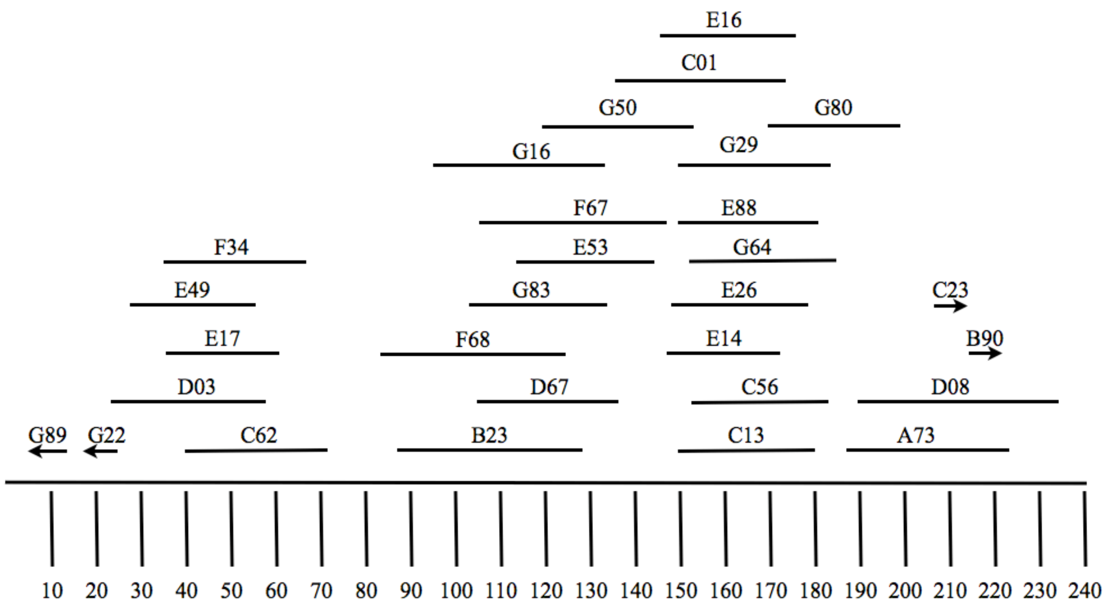

Figure S6. Within-contigs synteny between P1 and P2. Pairwise NUCmer comparison of all contigs > 5kbp from P2 against P1. The Dot Plot was generated with the mummerplot tool and the "--fat" option, which tries to order the contigs from both files so that the best alignment across all contigs is achieved. The Plot shows no indication of major within-contigs synteny breaks.

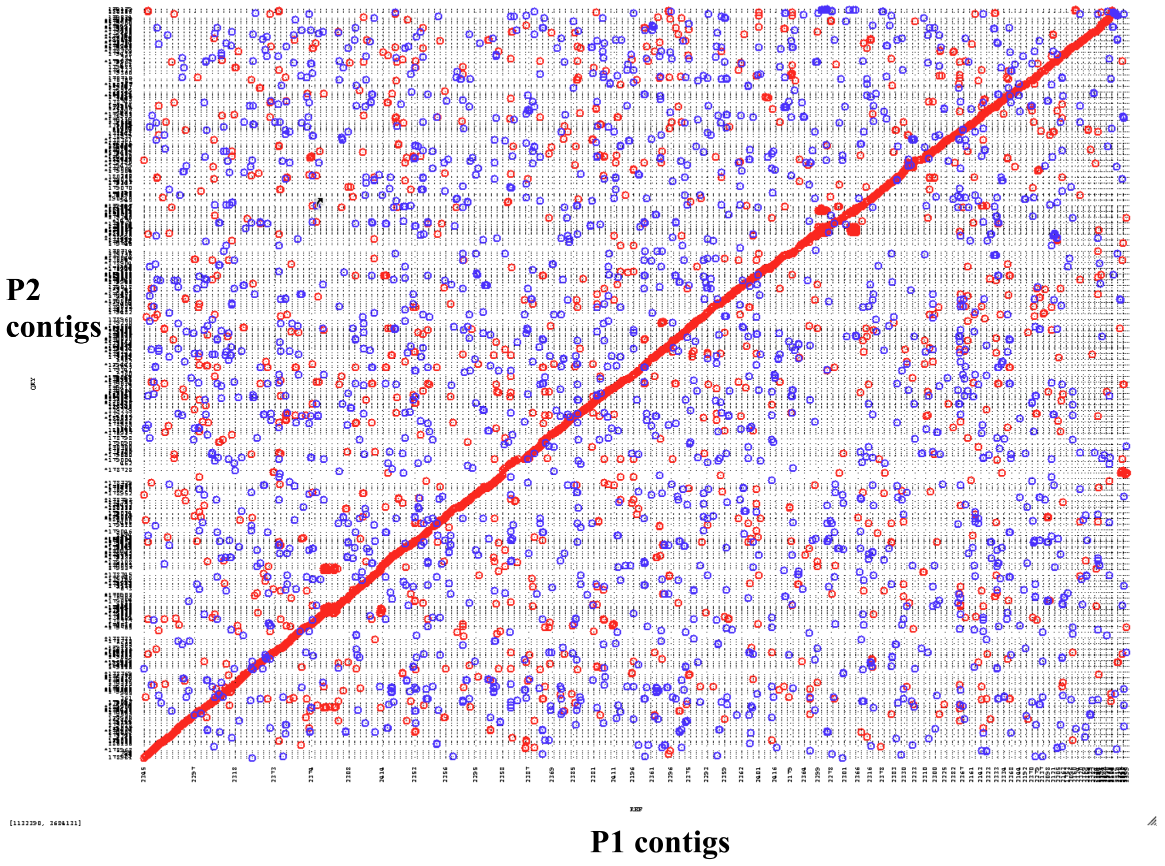

Figure S7. Within-contigs synteny between P1 and P3. Pairwise NUCmer comparison of all contigs > 5kbp from P3 against P1. The Dot Plot was generated with the mummerplot tool and the "--fat" option, which tries to order the contigs from both files so that the best alignment across all contigs is achieved. The Plot shows no indication of major within-contigs synteny breaks.

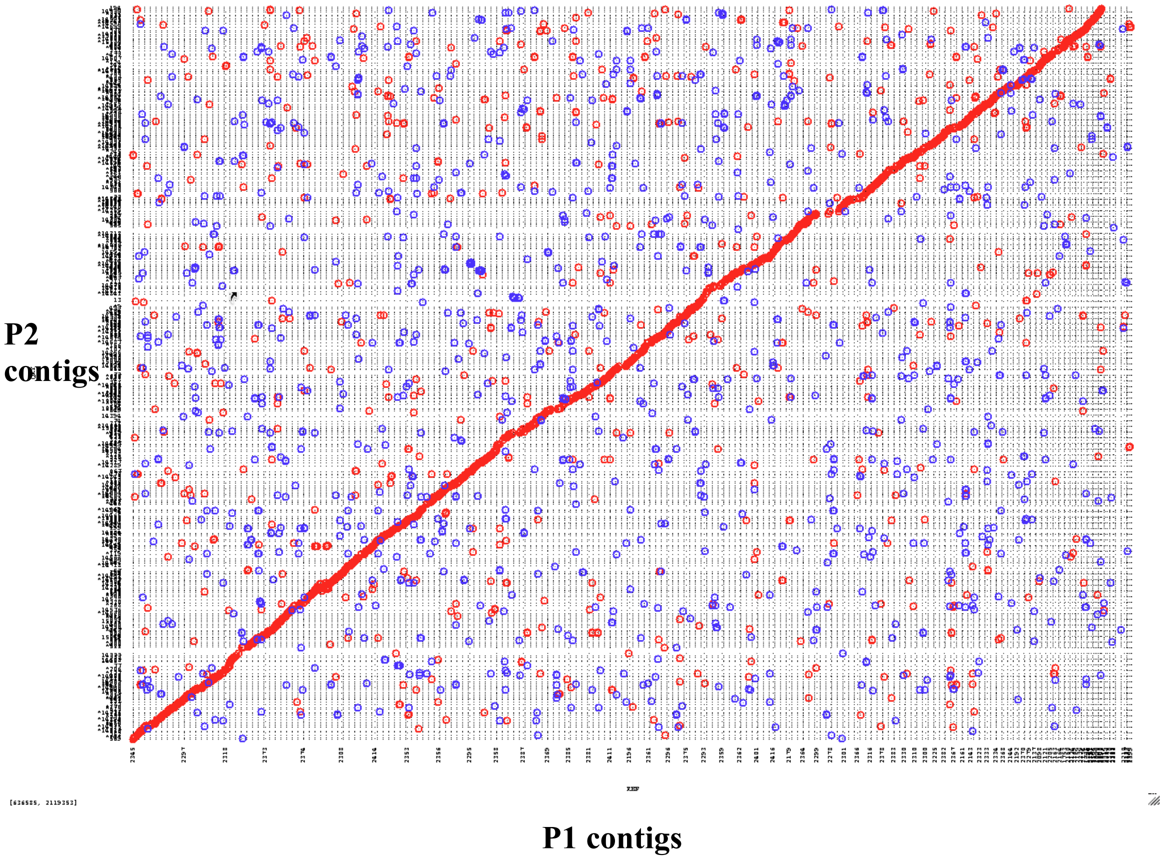

Figure S8. Organization of *prn*. NT, nucleotide transferase; ST, sulfotransferase; CT, carbamoyltransferase; green, oxidoreductases; yellow, radical SAM containing protein; blue, glycosyltransferases.

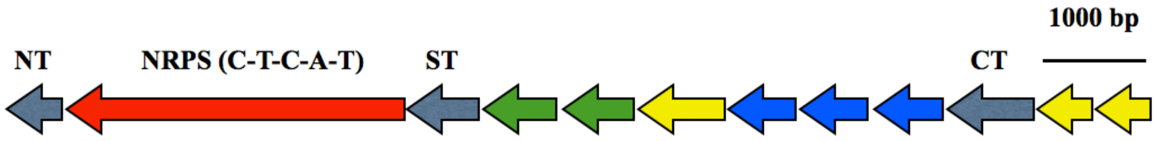

Figure S9. Organization of *nkd*. P, peptidase; H, hypothetical proteins; PT, prenyltransferase; PE, phosphoesterase; A, adenylation domain; MT, methyltransferase; C, condensation domain; T, thiolation domain; R, recombination protein; blue, gene products involved in the biosynthesis of the nucleoside moiety; yellow, ABC transporter. The predicted *nkd* product is also shown where R represents the multiple modifications that can take place (hydroxylation, prenylation, methylation).

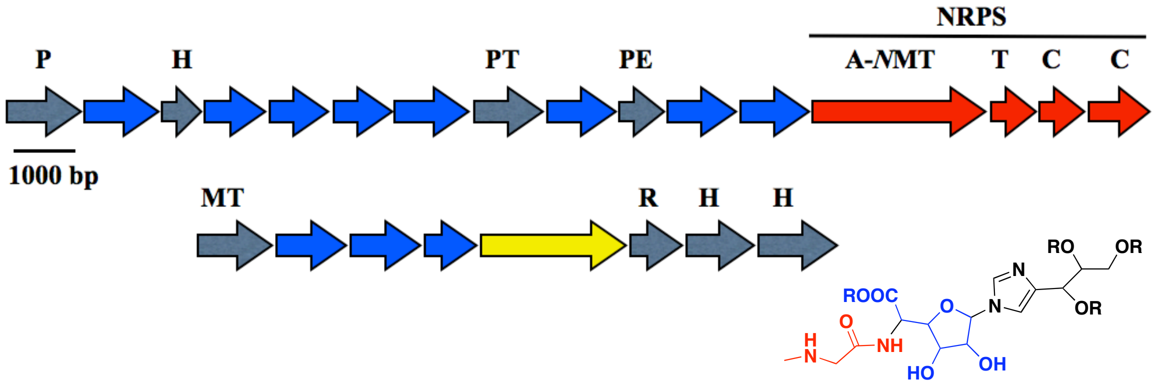

Figure S10. Organization of *pyr*. A, adenylation domain; DH, proline dehydrogenase; T, thiolation domian; Hyd, hydrolase / esterase domain; blue, halogenases; yellow, permease. Predicted products are shown where R is unknown and X might be either Cl or Br.

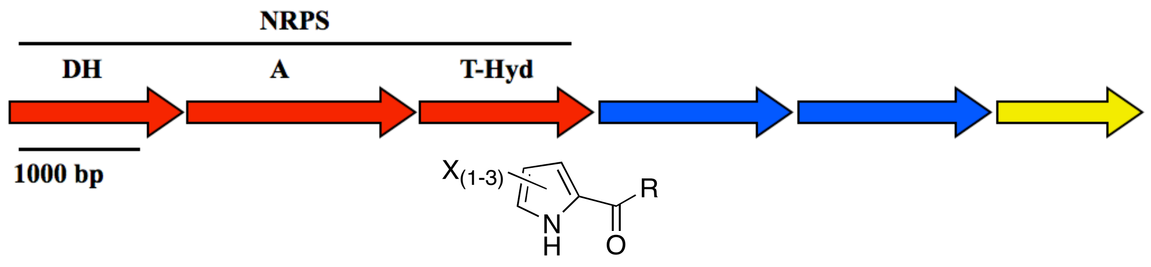

Figure S11. Organization of *pks*. KS, ketosynthase domain; AT, acyltransferase; ACP, acyl carrier protein; TE, thioesterase domain; T, transposase; *O*MT, *O*-methyl transferase; blue, sugar modifying proteins; yellow, ABC transporter.

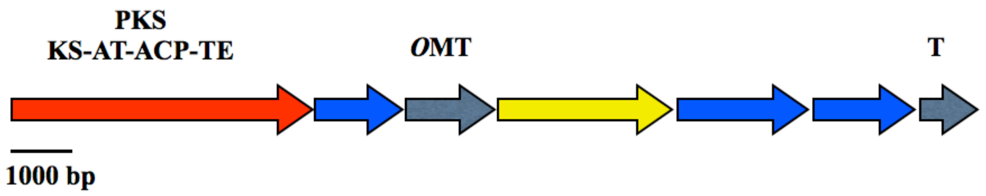

Figure S12. Organization of *gaz*. AL, acyl ligase; KS, ketosyanthase domain; AT, acetyl transferase; ACP, acyl carrier protein; KR, ketoreductase domain; ST, sulfotransferase; Hyd, hydrolase / esterase domain.

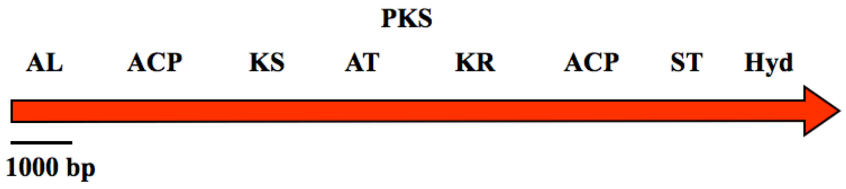

Figure S13. Genetic organization of *pat* and *tru*. Both pathways are ~11 kbp in length. N-pro, N terminus protease; DUF, domain of unknown function; Het, heterocyclization domain; YcaO, probable docking domain; Oxi, oxidase domains; C-pro cyc, C terminus protease and macrocyclase domain; E, precursor peptide.

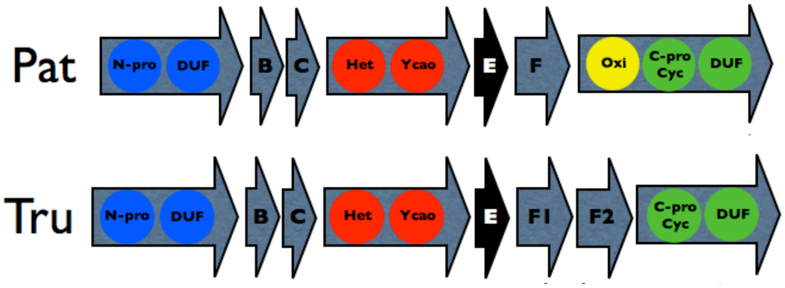

Figure S14. Comparison between *pat* and *tru*. Artemis Comparison Tool is used to visualize the nucleotide comparison between the *pat* and *tru* pathways. Percent identity is indicated on each segment. An example of a resulting cyanobactin is show on the right of each pathway.

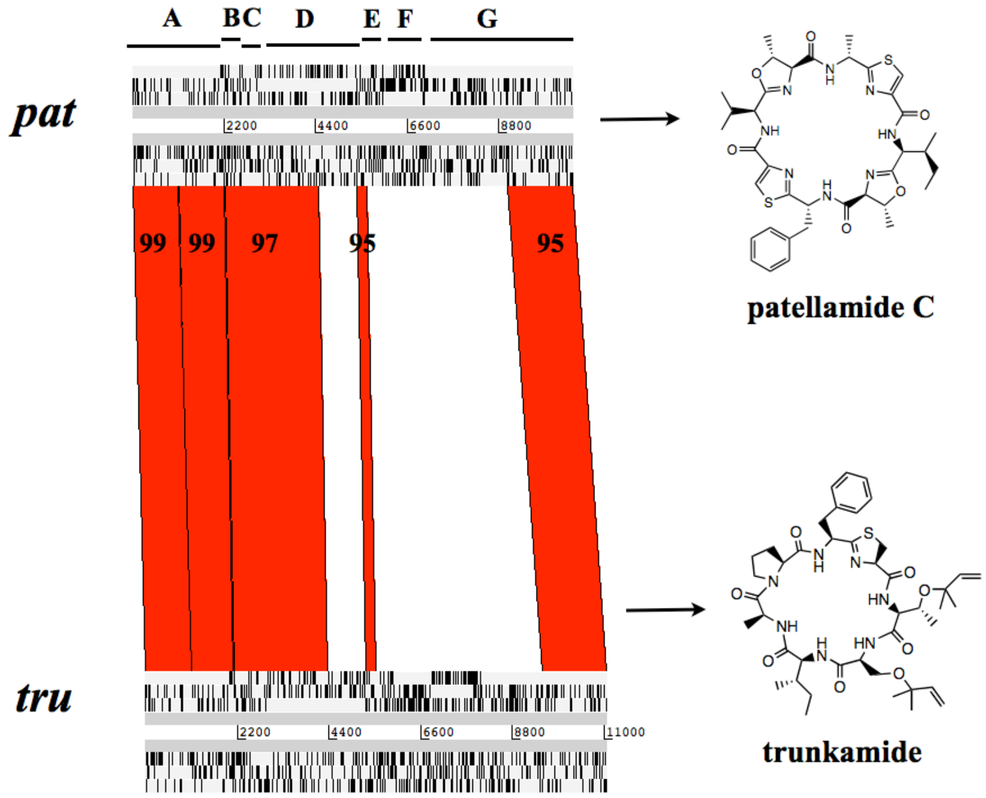

Figure S15. Functional cassettes in cyanobactin precursor peptides. An alignment of the amino acid sequence of the two cyanobactin precursor peptides from P1 (PatE1 and PatE2) and the precursor peptides from P2 (TruE1) and P3 (TruE2). The mutations accumulate only in the regions directly encoded for the final products (underlined) while the rest of the peptide remains constant.

```
PatE1: MNKKNILPQQGQPVIRLTAGQLSSQLAELSEEALGDAGLEASVTACITFCAYDGVEPSITVCISVCAYDGE
PatE2: MNKKNILPQQGQPVIRLTAGQLSSQLAELSEEALGDAGLEASVTACITFCAYDGVEPSCTLCCTLCAYDGE
TruE1: MNKKNILPQLGQPVIRLTAGQLSSQLAELSEEALG--GVDASTLPVPTLCSYDGVDAS-T-VPTLCSYDD
TruE2: MNKKNILPQLGQPVIRLTAGQLSSQLAELSEEALG--GVDASTFPVPTVCSYDGVDAS-TSIAPFCSYDD
       ********* *************************   *::**        * ****: * *     * **
```

Figure S16. Gene organization of *tom*. Cyc, cyclodehydratase; YcaO, docking domain; Ox, oxidase; Red, precursor peptide; Yellow, transporter; I, integrase.

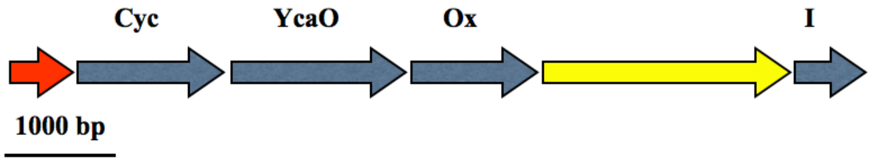

Figure S17. Predicted precursor peptides for *tom*. The cysteine-rich predicted precursor peptide from P1 and P2 are aligned. The cysteine-rich segment of SagA encoding for streptolysin A is shown for comparison.

```
Prochl1        MCIVDLSSLPLLGEIQMAKPEYSQLSANYPDFNNSIISLLEEGGGDPSILTPAAKDLLEG
Prochl2        MCIMDLSSLLLLGEIQMAKLEHSQLAANYPDLGNSIISLLEEGAGHASILTSVAKDLLEG
               ***:***** ********* *:***:*****:.**********.*..****..*******

Prochl1        DLVALAWHNNTERTEKLSVRDIYSIEEAFRTHGFYGSPD-EPLATACCCCTPACCCCAAA
Prochl2        DLLALAWHNNTERTEDLTVRDINSIEEAFCTDGFYGSPTGEPLAVACCCSFACCCCTASA
               **:************.*:**** ****** *.******  ****.****. ..*** *:*

Prochl1        LVDTVS                                   sagA---------CCCCCSCCCCVSAS---
Prochl2        VVDTVS
               :*****
```

Figure S18. Gene organization of the *hyd*. Cyc, cyclodehydratase; YcaO, docking domain; AH, amidohydrolase; Red, precursor peptide; JamD, halogenase.

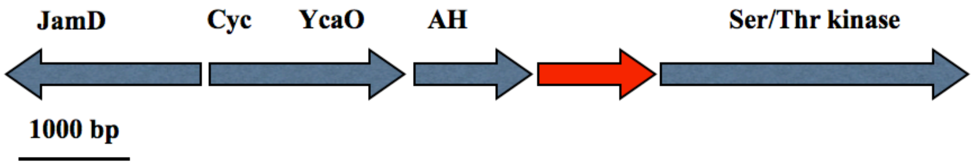

Figure S19. Predicted precursor peptides for *hyd*. Predicted precursor peptides form the amidohydrolase gene cluster in P1 and P2 are aligned. While the rest of the cluster proteins are almost identical, hypervariable regions can be observed in the precursor peptides.

```
Prochl1  241   VATQIEMIQPGKMQRPGHYEITMPYPPRPSSEDTTTTPDQVYSWAYADLYEMPGIENPFP  300
Prochl2  241   VATQIEMIQPGKMQRPGHYETTMPYPPRPSSEDTTTTPDQVYSWAYADLYDKDENPFPFY  300
               ******************** *****************************       **

Prochl1  301   FYSPCDEN------NKFVSTFVGTTYPPFPYIPVSTC  331
Prochl2  301   PPKKPEQNDLPPDENRFVSRFVGTTYPPFPYIPVSTC  337
                      *      * *** *****************
```

Figure S20. Gene organization of the terpene gene cluster identified in P1, P2 and P3. OC, oxysqualene cyclase; LS, lanosterol synthase; H, hypothetical protein; AD, aldehyde dehydrogenase; DH, NAD dependent dehydratase; SE, squalene epoxidase; SS, squalene synthase.

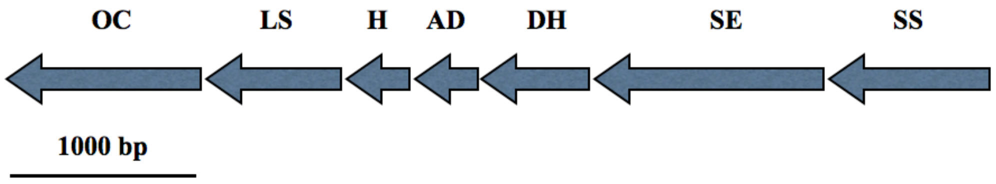

Figure S21. Strategy for PCR analysis of presence or absence of a pathway. Left, ACT was used to compare the genotypes of P1, P2 and P3 in regard to the *pyr* gene cluster. While P1 lacks the pathway, an exact insertion site can be observed in P2 and P3 which is flanked by genes conserved in all three genomes. Our PCR strategy was based on these flanking conserved regions where primers were designed to amplify across the insertion point. Therefore, the absence of the pathway will still give a positive PCR result as shown on the right. In addition, another pair of primers was designed to amplify within the gene cluster to confirm its presence. Stars indicate amplified bands of the correct size.

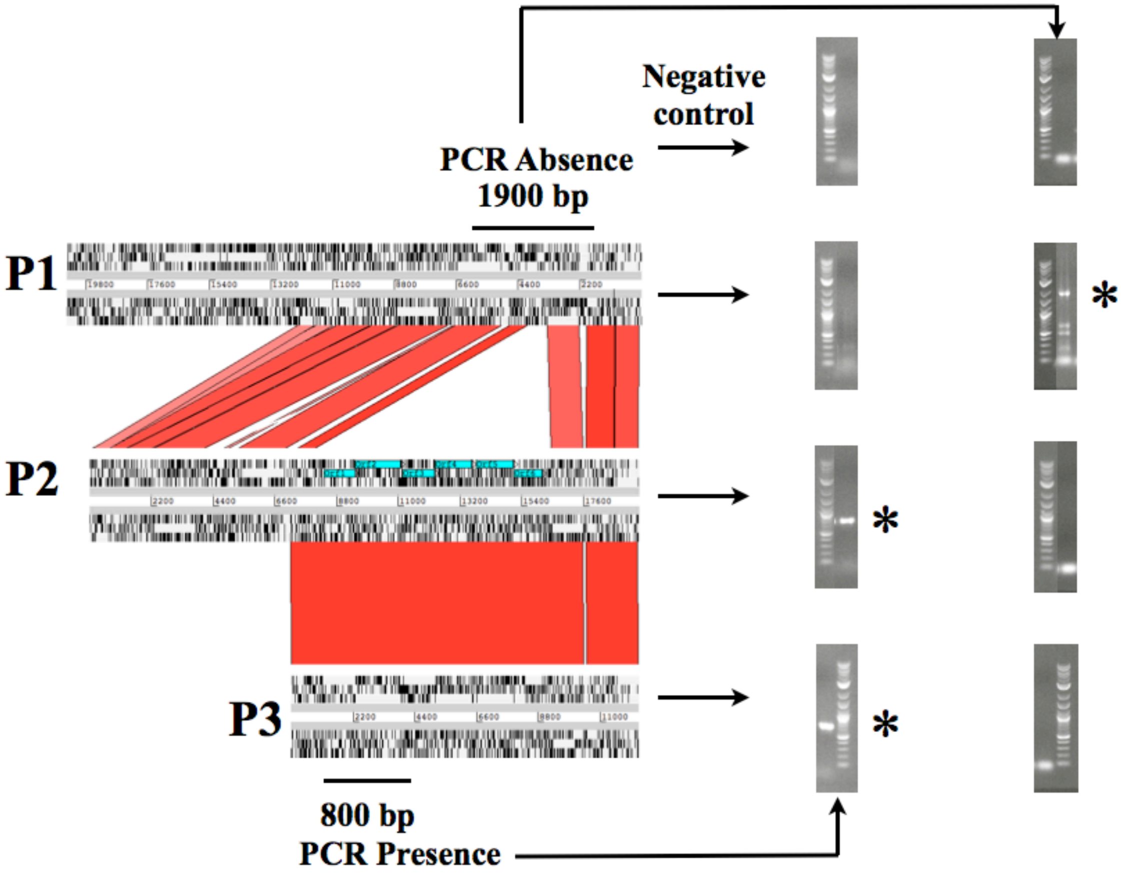

Figure S22. Strategy for PCR analysis of presence or absence of *nkd* pathway. ACT was used to compare the genotypes of P1 and P3 in regard to the *nkd* gene cluster. While P1 lacks the pathway, an exact insertion site can be observed in P3 ,which is flanked by genes conserved in the two genomes. Our PCR strategy was based on these flanking conserved regions where primers were designed to amplify across the insertion point. Two pairs of primers were used for the absence PCR and two pairs were used for the presence PCR.

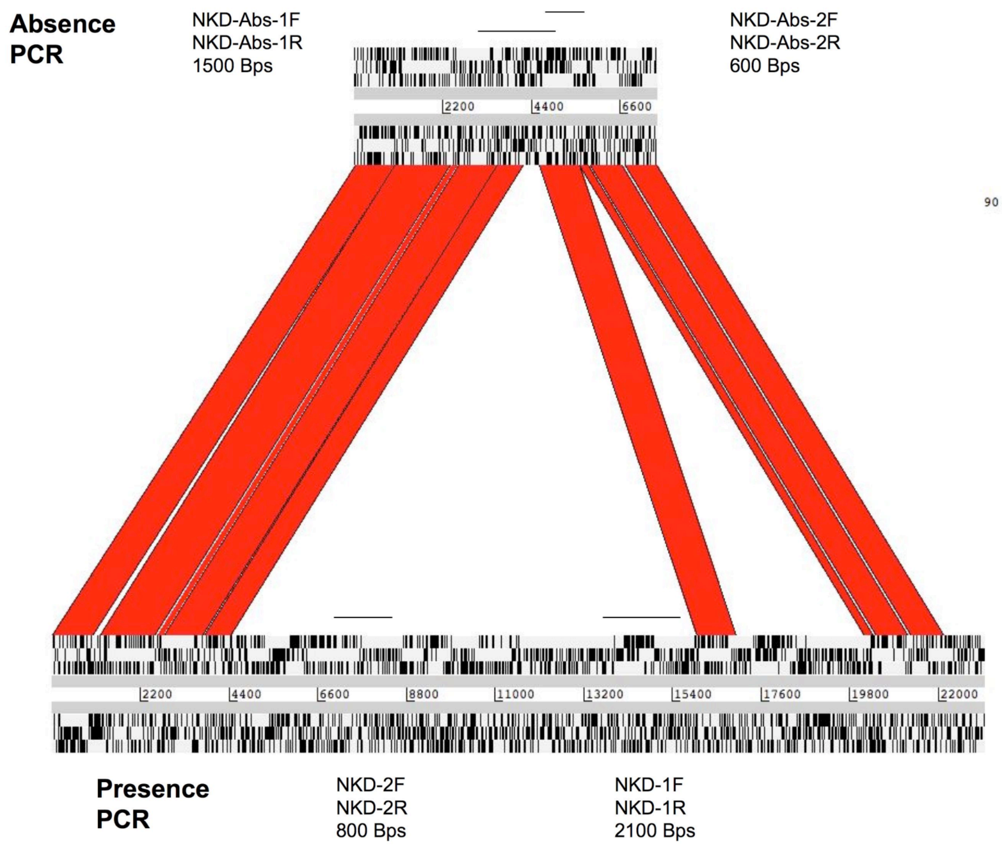

Figure S23. New TruE variants discovered by PCR. Alignment of two *tru* precursor peptides identified from *D. molle* samples 03-011 and 03-002, not know previously to contain prenylated cyanobactins. The sequence of the predicted products is highlighted in yellow.

03-11

N_{17}TAGQLSSQLAELSEEALGGVDASTLSPFCSYDGVDASIVPPFCSYDD

03-002

N_{17}TAGQLSSQLAELSEEALGGVDASTLSPFCSYDGVDASTTVTACSYDD

Figure S24. LC-FTMS analysis of *D. molle* 03-002. Top, LC-FTMS trace filtered to the mass of 699.3, corresponding to new cyanobactin mollamide E (**2**) predicted from the biosynthetic gene sequence. Bottom, accurate mass of the predicted cyanobactin.

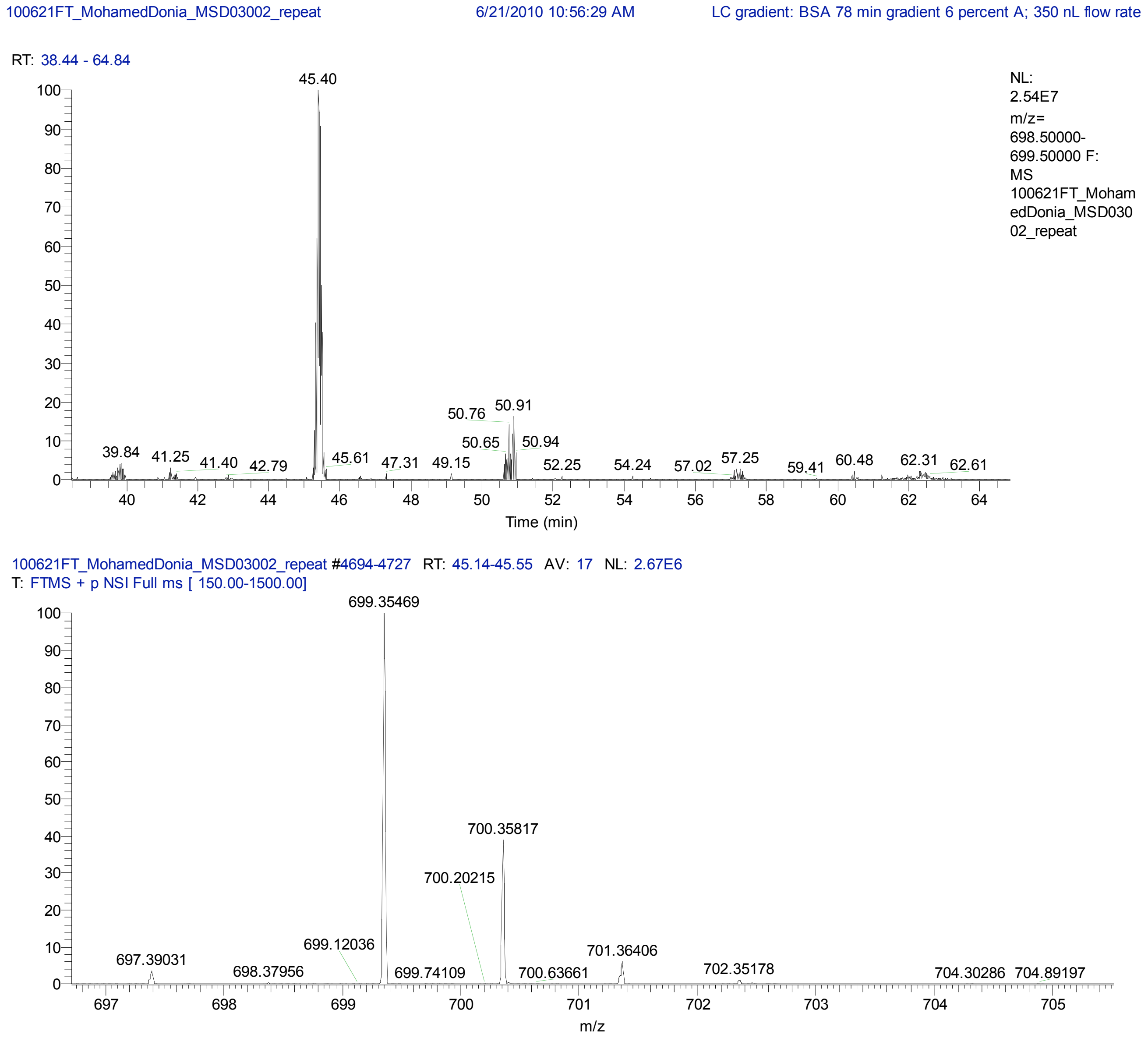

Figure S25. LC-FTMS analysis of *D. molle* 03-002. Top, LC-FTMS trace filtered to the mass of 763.4, corresponding to new cyanobactin mollamide D (**1**) predicted from the biosynthetic gene sequence. Bottom, accurate mass of the predicted cyanobactin.

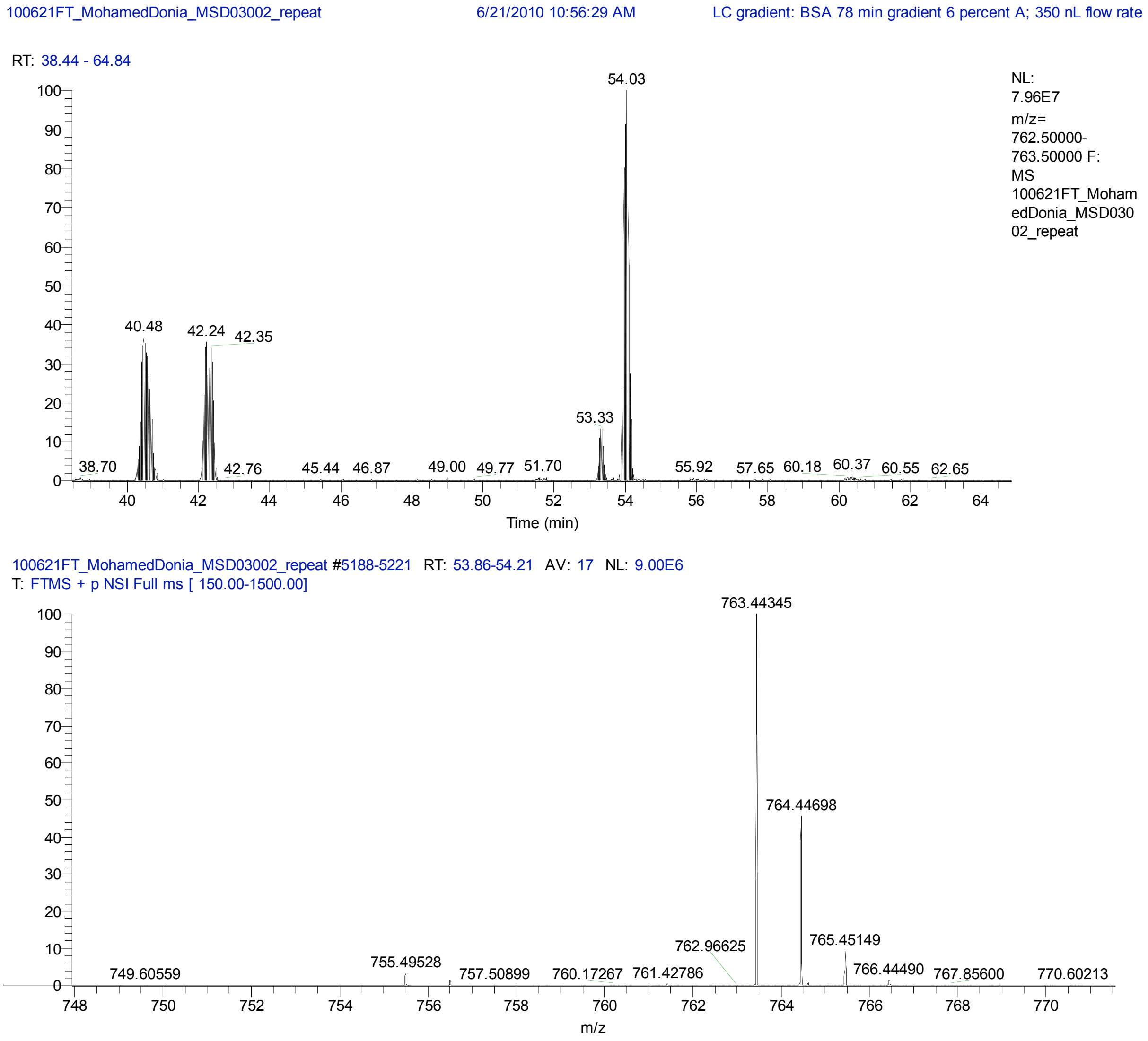

Figure S26. FT-MSMS analysis of three prenylated cyanobactins.

Left, structure of cyanobactins. Middle, accurate mass. Right, ms^2 showing the consecutive loss of prenyl groups. All accurate masses are within 0.2-2.8 ppm of the calculated mass (errors in ppm are shown with each observed mass). Top, trunkamide (**3**). Middle, mollamide D, TTVTAC (**1**). Bottom, mollamide E, TLSPFC (**2**).

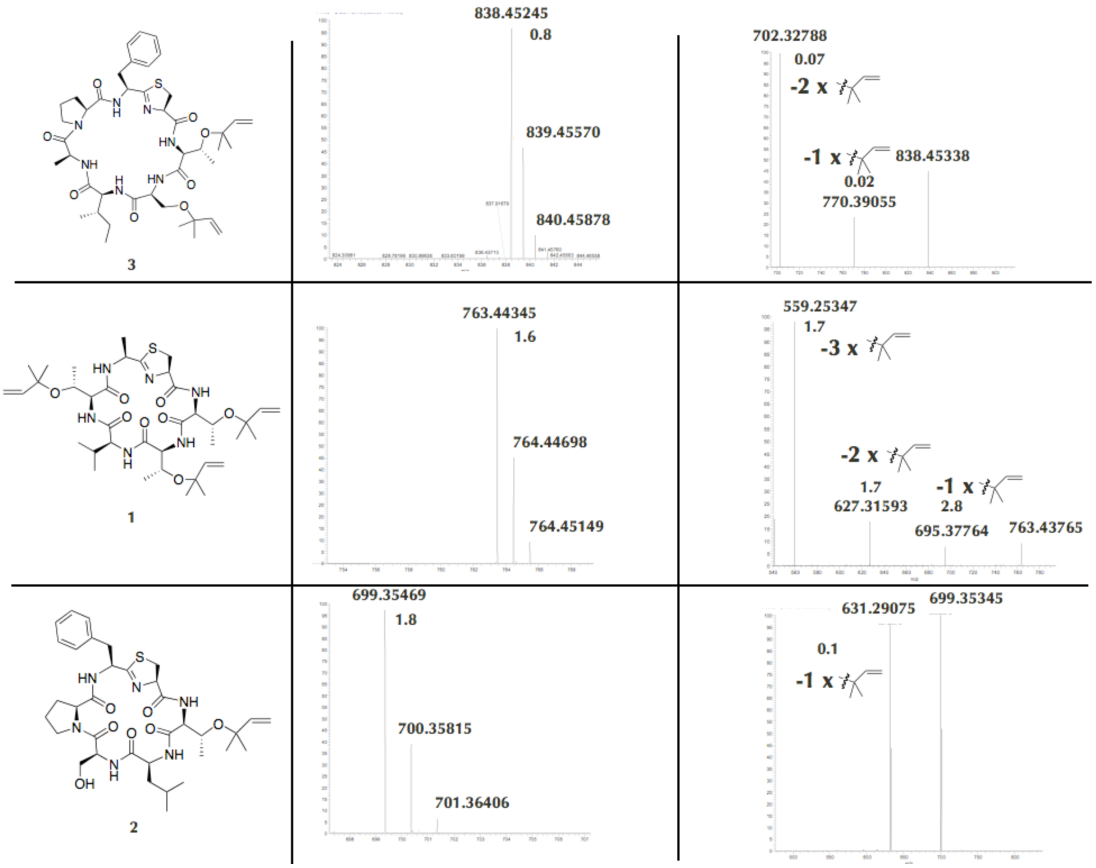

Table S1. Genome Statistics

	P1	P2	P3
Total No. of Contigs[1]	216	3,285	1,334
Sequencing	Sanger / 454/ Fosmid	454 / Fosmid	454
Libraries	Sanger: small (5-8 kbp) and medium (12-15 kbp) plasmid, large (30-50 kbp) fosmid 454: single-read GS	Sanger: large (30-50 kbp) fosmids 454: single-read GS FLX Titanium	454: single-read GS FLX Titanium
Contigs assigned to *P. didemni*[1]	98	869	1,093
Contigs >25 kbp (No. / length)	57 / 5,777,450 bp	44 / 1,535,704 bp	10 / 307,018 bp
Largest contig	316,282 bp	84,370 bp	40,850 bp
Total sequence length[3]	6,035,098 kbp	6,005,315 kbp	5,267,607 bp
GC content	41.9 %	41.8 %	41.9 %
Average coverage[2]	12x	90x	14x

[1]Larger than 1 kbp. [2]Based on 10 largest contigs. [3]Based upon contigs larger than 1 kbp, whereas the size in the methods section is based upon all reads.

Table S2. Collection information for samples used in this study. Bold indicates genome-sequenced sample.

Year	Code	Collection Site	Species	Location
**2002**	**Reef**	**Palau**	***L. patella***	**N 7º15' E 134º15'**
2003	03-001	PNG-Madang	*L .patella*	S 5º10.160' E 145º50.291'
2003	03-002	PNG-Madang	*D. molle*	S 5º10.160' E 145º50.291'
2003	03-005	PNG-Madang	*L. patella*	S 5º7.288' E 145º49.238'
2003	03-009	PNG-Madang	*D. molle*	S 4º47.290' E 146º11.796'
2003	03-011	PNG-Madang	*D. molle*	S 4º46.75' E 146º11.16'
2003	03-012	PNG-Madang	didemnid	S 4º46.75' E 146º11.16'
2003	03-017	PNG-Madang	*L. patella*	S 4º44.942' E 145º58.310'
2003	03-018	PNG-Madang	*Diplosoma*	S 4º44.942' E 145º58.310'
2003	03-019	PNG-Madang	didemnid	S 4º44.942' E 145º58.310'
2003	03-020	PNG-Madang	*Diplosoma*	S 4º44.942' E 145º58.310'
2005	05-019	PNG-MilneBay	*L. patella*	S 10º15.856' E 150º15.856'
2005	05-031	PNG-MilneBay	didemnid	S 10º12.614' E 150º34.848'
2005	05-032	PNG-MilneBay	didemnid	S 10º12.614' E 150º34.848'
2005	05-042	PNG-MilneBay	didemnid	S 10º15.502' E 150º46.889'
2006	06-008	Solomons	*Lissoclinum* sp.	S 9°1.02' E 158°43.83'
2006	06-014	Solomons	Ascidian	S 9º0.13' E 159º15.69'
**2006**	**06-027**	**Solomons**	***L. patella***	**S 8°57.35' E 159°59.12'**
2006	06-029	Solomons	*Lissoclinum* sp.	S 8°57.35' E 159°59.12'
2006	06-030	Solomons	*Lissoclinum* sp.	S 8°57.35' E 159°59.12'
**2006**	**06-037-2**	**Fiji**	***L. patella***	**S 17º55' E 177º16'**
2007	07-001	Palau	*Diplosoma*	N 7°19'22.39" E 134°29'8.33"
2007	07-005	Palau	*L. patella*	N 7°18'45.69" E 134°13'28.96"
2007	07-021	Palau	*Lissoclinum* sp.	N 7°21'0.40" E 134°34'29.08"

Table S3. Primers used in this study.

Primer	Purpose	Sequence
Pyr-1F	*pyr* presence	GTTGGCTGATGGATCAAAGTCTG
Pyr-1R	*pyr* presence	TGTCTCTTGAACGTCTGAAGGC
Pyr-Abs-1F	*pyr* absence	CATGAAAGGACAAGTGAGTCGGG
Pyr-Abs-1R	*pyr* absence	CTCAGTCTTCTGTAACTCCTCC
NKD-1F	*nkd* presence	GGTCGATTGGCAAATAGAGCAAC
NKD-1R	*nkd* presence	GTCGTCTGCTATTGTACTCATTTGC
NKD-2F	*nkd* presence	CTGGTGTGCGAAAGCAGGAATC
NKD-2R	*nkd* presence	GATGGCAGCAAGTATTGAGGCAAC
NKD-Abs-2F	*nkd* absence	CCTGTTCTAGTAGGTCGCTTAC
NKD-Abs-2R	*nkd* absence	CGGTCCTATTCCTGGTAACCGT
NKD-Abs-1F	*nkd* absence	GGAACTGCCAGCATATATATGAAGC
NKD-Abs-1R	*nkd* absence	CAAAGATGTGCCATCGCGTTCC
PatAf-Bsph1	*pat/tru* presence	ATCATGAATAGAGATATTTTGCGAAC
PatAr-350	*pat/tru* presence	CGCCGCTGATGTTGATAATGTG
Cao-F	*Prochloron* presence	CAACCCCTATGCCTTTTGAA
Cao-R	*Prochloron* presence	TAAACAACCCATGCTCCACA
patEf-NoRes	*pat/tru presence*	ATGAACAAAAAAAACATTCTGCCCCAA
TruE-R4	*tru* presence	CTCGCTAGATTAGTCGTCGTAAG
PatE-R-Kpn1	*pat* presence	TTCTTCTTGGTACCCTTATTCACCATC
TruF1-R-Kpn1	*tru* presence	TTTGGTACCCTATACCGCCTTGCGATAATAG
PatF-R-Kpn1	*pat* presence	ATGACTAGGTACCTGAGTCAATGCAAATG

Table S4. Predicted open reading frames in the three identified nonribosomal gene clusters.

Cluster	Orf number	Predicted function	Closest homolog<br>(% Identity protein level)
prn	Orf1	Nucleotidyl transferase	Hypothetical protein (57%)<br>*Microcoleus chthonoplastes* PCC 7420
	Orf2	NRPS	NRPS (52%)<br>*Anabaena variabilis* ATCC 29413
	Orf3	Sulfotransferase	Sulfotransferase (27%)<br>*Maricaulis maris* MCS10
	Orf4	Oxidoreductase	Oxidoreductase (39%)<br>*Clostridium acetobutylicum* ATCC 824]
	Orf5	(Fe-S) oxidoreductase	(Fe-S) oxidoreductase (25%) *Streptomyces tenjimariensis*
	Orf6	Radical SAM	Radical SAM (24%)<br>*Clostridium papyrosolvens* DSM 2782
	Orf7	Glycosyl transferase	Glycosyl transferase (36%)<br>*Geobacillus kaustophilus* HTA426
	Orf8	Glycosyl transferase	Glycosyl transferase (36%)<br>*Geobacillus kaustophilus* HTA426]
	Orf9	Glycosyl transferase	Glycosyl transferase (36%)<br>*Geobacillus kaustophilus* HTA426]
	Orf10	Carbamoyltransferase	Carbamoyltransferase (34%)<br>*Magnetococcus* sp. MC-1
	Orf11	Radical SAM	Radical SAM (54%)<br>*Actinosynnema mirum* DSM 43827
	Orf12	Radical SAM	Radical SAM (43%)<br>*Actinosynnema mirum* DSM 43827
pyr	Orf1	L-prolyl-S-PCP-Dehydrogenase	AcylCoA dehdrogenase<br>*Myxococcus xanthus* (57%)
	Orf2	NRPS<br>Proline adenyltransferase	Linear gramicidin synthetase<br>*Stigmatella aurantiaca* (55%)
	Orf3	NRPS<br>PCP-Esterase/Hydrolase	Hydrolase<br>*Meiothermus rubber* (30%)

	Orf4	Halogenase	Halidase<br>*Planctomyces maris* (29%)
	Orf5	Halogenase	Halogenase<br>*Plesiocystis pacifica* (50%)
	Orf6	Permease	Permease<br>*Beggiatoa sp.* (54%)
nkd	Orf1	Peptidase<br>L-acyl amino acid hydrolase	L-acyl amino acid hydrolase (75%)<br>Lyngbya sp. PCC 8106
	Orf2	Imidazole glycerol phosphate synthase	Imidazole glycerol phosphate synthase (84%)<br>*Cyanothece* sp. CCY0110
	Orf3	Hypothetical protein	Hypothetical protein (78%)<br>*Cyanothece* sp. PCC 8801
	Orf4	UDP-N-acetylglucosamine 1-carboxyvinyltransferase	NikO 30%<br>*Streptomyces tendae*
	Orf5	UDP-N-acetylglucosamine 1-carboxyvinyltransferase	UDP-N-acetylglucosamine 1-carboxyvinyltransferase (41%)<br>*Photorhabdus asymbiotica*
	Orf6	UDP-N-acetylglucosamine 1-carboxyvinyltransferase	UDP-N-acetylglucosamine 1-carboxyvinyltransferase (41%)<br>*Photorhabdus asymbiotica*
	Orf7	Radical SAM	NikJ (46%)<br>*Streptomyces tendae*
	Orf8	UbiA like prenyltransferase	hypothetical protein (32%)<br>*Micromonospora* sp. ATCC 39149
	Orf9	Nucleoside triphosphate hydrolases	Topology modulation protein(52%)<br>*Bacillus mycoides* Rock3-17
	Orf10	Phosphoesterase	phosphoesterase (41%)<br>*Nostoc punctiforme* PCC 73102
	Orf11	Hydroxylase	NikM (29%)<br>*Streptomyces tendae*
	Orf12	tyrosine/serine phosphatase	NikL (29%)<br>*Streptomyces tendae*
	Orf13	NRPS (A-*N*MT)	non-ribosomal peptide synthetase (48%)<br>*Anabaena* sp. 90
	Orf14	NRPS (T)	amino acid adenylation domain protein (44%)<br>*Cyanothece* sp. PCC 7424

	Orf15	NRPS (C)	Amino acid adenylation domain-containing protein (39%) *Nostoc punctiforme* PCC 73102
	Orf16	NRPS (C)	Amino acid adenylation domain-containing protein (33%) *Nostoc punctiforme* PCC 73102]
	Orf17	Methyltransferase	Methyltransferase type 11 (43%) *Herpetosiphon aurantiacus* ATCC 23779
	Orf18	Histidinol-phosphate aminotransferase	NikK (25%) *Streptomyces tendae*
	Orf19	Histidinol-phosphate aminotransferase	NikK (26%) *Streptomyces tendae*
	Orf20	Hydroxylase	NikM (42%) *Streptomyces tendae*
	Orf21	ABC transporter	Cyclic peptide transporter (48%) *Nostoc punctiforme* PCC 73102
	Orf22	Recombination protein	Recombination protein RecR (88%) *Microcoleus chthonoplastes* PCC 7420
	Orf23	Hypothetical protein	Hypothetical protein Avi_7136 (27%) *Agrobacterium vitis* S4
	Orf24	Hypothetical protein	Protein of unknown function DUF820 (48%) *Cyanothece* sp. PCC 7424

Table S5. Predicted open reading frames in the two identified polyketide synthase gene clusters.

Cluster	Orf number	Predicted function	Closest homolog<br>(% Identity protein level)
prn-pks	Orf1	PKS<br>(KS-AT-ACP-TE)	Polyketide synthase (46%) *Beggiatoa* sp. PS
	Orf2	N-acetylhexosaminidase	Beta-N-acetylhexosaminidase (71%)<br>*Trichodesmium erythraeum* IMS101
	Orf3	Methyltransferase	*O*-methyltransferase family 2 (33%)<br>*Beggiatoa* sp. PS
	Orf4	ABC transporter	ABC transporter related (53%)<br>*Trichodesmium erythraeum* IMS101
	Orf5	Carbamoylphosphate synthase	Hypothetical protein (41%)<br>*Clostridium acetobutylicum* ATCC 824
	Orf6	Diacylglycerol glucosyltransferase	Hypothetical protein (41%)<br>*Clostridium acetobutylicum* ATCC 824
	Orf7	Transposase	ProF (79%) *Planktothrix rubescens*
gaz	Orf1	PKS<br>(AL-ACP-KS-AT-KR-ACP-ST-Hyd)	Polyketide synthase (51%)<br>*Cyanothece* sp. PCC 7424

Table S6. Predicted open reading frames for *hyd and* tom clusters identified in *Prochloron*.

Cluster	Orf number	Predicted function	Best blast hit
tom	Orf1	Precursor peptide	None
	Orf2	Cyclodehydrartae	*Rhodobacterales* bacterium HTCC2083 (23%)
	Orf3	Ycao	*Rhodobacterales* bacterium HTCC2083 (37%)
	Orf4	Flavin oxidoreductase	*Rhodobacterales* bacterium HTCC2083 (38%)
	Orf5	Transporter	*Rhodobacterales* bacterium HTCC2083 (27%)
hyd	Orf1	JamD halogenase / oxidase	*Lyngbya majuscula* (51%)
	Orf2	Cyclodehydratase-YcaO	*Cyanothece* sp. PCC 7822 (68%)
	Orf3	Amidohydrolase	*Cyanothece* sp. PCC 7822 (67%)
	Orf4	Precursor peptide	*Cyanothece* sp. PCC 7822 (27%)
	Orf5	Serine / threonine kinase	*Cyanothece* sp. PCC 7424 (64%)

Table S7. Predicted open reading frames in the terpene gene cluster (*ter*) from *Prochloron*.

Orf number	Predicted function	Best blast hit
Orf1	Squalene and phytoene synthases	*Synechococcus* sp. PCC 7335 (63%)
Orf2	Squalene epoxidase	*Stigmatella aurantiaca* DW4/3-1 (27%)
Orf3	NAD-dependent epimerase/dehydratase	*Plesiocystis pacifica* SIR-1 (41%)
Orf4	Aldehyde dehydrogenase	*Lyngbya* sp. PCC 8106] (61%)
Orf5	Hypothetical protein	None
Orf6	Lanosterol synthase	*Rattus norvegicus* (35%)
Orf7	Squalene cyclase	*Methylococcus capsulatus* str. Bath (41%)
